# Light modulates organ-specific gibberellin–abscisic acid crosstalk during drought response in *Arabidopsis thaliana*

**DOI:** 10.1007/s00425-026-05204-7

**Published:** 2026-10-17

**Authors:** Tereza Miksteinová, Marek Szecówka, Ivan Petřík, Terezie Urbanová, Tereza Trávníčková, Martina Špundová, Martin Fellner, Peter Hedden

**Affiliations:** 1 Laboratory of Growth Regulators, Institute of Experimental Botany, Czech Academy of Sciences, Šlechtitelů 27, 77900 Olomouc, Czech Republic; 2 Laboratory of Growth Regulators, Faculty of Science, Palacký University, Šlechtitelů 27, 77900 Olomouc, Czech Republic; 3 Centre of Plant Structural and Functional Genomics, Institute of Experimental Botany, Czech Academy of Sciences, Olomouc, Czech Republic; 4 Department of Biophysics, Faculty of Science, Palacký University, Olomouc, Czech Republic; 5 Rothamsted Research, Harpenden, AL5 2JQ UK

**Keywords:** Blue light, Drought stress, GA 2-oxidase, GA biosynthesis, GA catabolism, Hormone crosstalk, Stomatal regulation

## Abstract

**Main conclusion:**

Continuous blue light enhances drought tolerance in Arabidopsis by modulating ABA and GA pathways, whilst controlled GA catabolism, especially via GA2ox7/8, is important for stomatal regulation and drought adaptation.

**Abstract:**

In response to drought, plants undergo physiological and developmental adjustments to reduce water loss and survive the stress, including reduced shoot growth, altered root architecture, and stomatal closure. These processes are regulated by phytohormones, including gibberellins (GAs), which mediate growth adjustments, and abscisic acid (ABA), which promotes stomatal closure. In addition, light signalling plays a crucial role in plant adaptation to drought. Here, we investigated how progressive water restriction affects hormone levels, gene expression, and leaf temperature in *Arabidopsis thaliana* shoots and roots under two contrasting light regimes: white light under short-day and continuous low-intensity blue light, with a focus on GA and ABA signalling pathways. The hormonal contribution to the drought response was investigated by analysis of ABA and GA mutants. Plants grown under continuous blue light experienced less-drought stress compared with white light as reflected in growth performance and differential gene expression. Drought increased ABA levels in leaves and roots, with stronger accumulation in roots under both light conditions. By contrast, GA production was decreased in leaves and increased in roots under drought in white light, but with little change in blue light. The change in GA status in leaves was associated with down-regulation of GA-biosynthetic *20-OXIDASE* genes and up-regulation of GA-catabolic *2-OXIDASE* genes (*AtGA2ox6, AtGA2ox9*). ABA- and GA-deficient mutants were unaffected by water restriction, whilst GA-overproducers lacking *GA2ox* genes or overexpressing *AtGA20ox1* were hypersensitive to drought, with the C_20_-GA2ox genes *GA2ox7* and *GA2ox8* proving particularly critical with compromised stomatal regulation.

**Supplementary Information:**

The online version contains supplementary material available at https://doi.org/10.1007/s00425-026-05204-7.

## Introduction

Plants are subject to a variety of abiotic and biotic stresses, with drought one of the most prevalent and damaging, and a major constraint to crop production globally (Zhang et al. 2018). In response to drought, plants modify their physiology to reduce water demand by closing stomata and slowing shoot growth, whilst root growth may be maintained or even promoted, depending on the severity of the water restriction (Sharp et al. 2004; Gupta et al. 2020). Many of these effects are mediated by hormones, with abscisic acid (ABA), the synthesis of which is strongly induced by drought stress, regulating stomatal movement (Waadt et al. 2022). However, the involvement of hormones in regulating the growth redistribution between shoots and roots in response to water restriction is less clear. The growth hormone gibberellin (GA) is required for normal growth of shoots and roots although roots require lower concentrations of GA than do shoots (Tanimoto 2012). Consequently, a reduction in GA content impacts shoot growth more severely than that of roots, making this hormone a strong candidate for involvement in the growth redistribution that results from drought. Furthermore, in response to drought, in common with other abiotic stresses, plants reduce their GA content (Colebrook et al. 2014). 

The main biologically active GAs in vegetative tissues of higher plants are GA_4_ and its 13-hydroxy analogue GA_1_. They are biosynthesised from the common diterpenoid precursor *trans*-geranylgeranyl diphosphate, which is converted in two steps in plastids to the hydrocarbon *ent*-kaurene via *ent*-copalyl diphosphate (CPP), catalysed by *ent*-copalyl diphosphate synthase (CPS) and *ent*-kaurene synthase (KS). Oxidation of *ent*-kaurene to the C_20_-GA GA_12_ is catalysed by the cytochrome P450 monooxygenases *ent*-kaurene oxidase (KO) and *ent*-kaurenoic acid oxidase (KAO), situated on the plastid envelope and endoplasmic reticulum, respectively, whilst a third monooxygenase, GA 13-hydroxylase, converts GA_12_ to GA_53_. In the final stage of the pathway catalysed by soluble 2-oxoglutarate-dependent dioxygenases (2-ODDs) in the cytosol, GA_12_ and GA_53_ are converted to the C_19_-GAs GA_9_ and GA_20_, respectively, by GA 20-oxidases (GA20ox) and then to GA_4_ and GA_1_ by GA 3-oxidases (GA3ox). Two further clades of 2-ODDs convert GAs to 2β-hydroxylated products acting on C_20_-GAs (C_20_-GA2ox) or C_19_-GAs (C_19_-GA2ox). These products are biologically inactive or cannot be converted to an active end-product and their formation is an important mechanism for regulating GA concentration. *GA2ox* genes are up-regulated by stress as part of a mechanism to reduce GA concentration (Colebrook et al. 2014). *Arabidopsis thaliana* (Arabidopsis), for which GA_4_ is the predominant active form, possesses one copy of the early GA-biosynthesis genes, *CPS*, *KS* and *KO*, two redundant *KAO* genes, five *GA20ox,* four *GA3ox,* four *C*_*20*_*-GA2ox* and five functional *C*_*19*_*-GA2ox* genes (Hedden 2020). The multiple 2-ODD paralogues differ in their spatial and temporal expression profiles and regulatory mechanisms (Hedden and Thomas 2012). 

GAs act by destabilising DELLA proteins, which are transcriptional regulators that inhibit plant growth (Zentella et al. 2007). They act in partnership with transcription factors, whose activity they may suppress or promote (Shani et al. 2024). Interaction with GA causes its receptor GIBBERELLIN-INSENSITIVE DWARF 1 (GID1) to undergo a conformational change that promotes its association with DELLA proteins (Ueguchi-Tanaka et al. 2007; Murase et al. 2008; Shimada et al. 2008). The GID1–GA–DELLA complex is recognised by the F-box protein of an SCF E3-ligase, resulting in DELLA ubiquitination and subsequent degradation by the 26S proteasome (Sun 2011). Thus, a low GA environment allows DELLA proteins to accumulate and suppress growth. By interacting with components of multiple signalling pathways, including those of other hormones and light, DELLAs mediate crosstalk between these pathways and GA signalling. DELLAs interact with and suppress the activity of PHYTOCHROME INTERACTING FACTOR (PIF) transcription factors, which mediate red/far-red light signalling as substrates for phytochrome receptors (Wang et al. 2022). Furthermore, DELLAs are stabilised by interacting directly with the blue light receptor CRYPTOCHROME1 (CRY1) which contributes to light-induced growth reduction (Xu et al. 2021; Yan et al. 2021). This effect is reinforced by the interaction of CRY1 with GID1 which suppresses GA-mediated DELLA degradation. In contrast to their convergent roles in growth suppression, DELLAs and blue light have opposite functions in regulating stomatal movement, whereas DELLAs promote stomata closure by enhancing ABA action (Shohat et al. 2020; Wang et al. 2020b), blue light opens stomata via the CRY and PHOTOTROPIN (PHOT) receptors (Kinoshita et al. 2001; Mao et al. 2005).

The function of GAs in stress responses as well as in other physiological processes is antagonised by that of ABA (Liu and Hou 2018). ABA is a product of the carotenoid pathway in which the xanthophylls 9-*cis*-neoxanthin and 9-*cis-*violaxanthin are cleaved by 9-*cis*-epoxycarotenoid dioxygenases (NCED) to xanthoxin, which is oxidised to ABA via abscisic aldehyde, catalysed by ABA2 and abscisic aldehyde oxidase (AAO) (Nambara and Marion-Poll 2005). The main inactivation pathway for ABA involves 8'-hydroxylation by CYP707A enzymes, resulting, after rearrangement, in the formation of phaseic acid. Interaction of ABA with the PYR/PYL/RCAR receptors promotes their interaction with group A protein phosphatase type 2 C (PP2Cs), which are thereby inactivated and allow SNF1-related protein kinases (SnRK2) to be maintained in their phosphorylated active state (Chen et al. 2020). SnRK2s phosphorylate multiple targets to modify their activity, including ion channels in stomatal guard cells (Osakabe et al. 2013). ABA and light signalling are integrated in stress responses including regulation of root growth, stomatal development and movement (Mukherjee et al. 2013).

Here we report the effects of progressive drought on hormone and transcript abundance in Arabidopsis shoots and roots growing under two contrasting light regimes: white-light short-day (SD) and continuous low-intensity blue light, with a focus on GA and ABA signalling pathways. Light quality represents an important factor that can modify growth and influences the interpretation of drought-induced responses (Christie 2007; Jiao 2007). Continuous low-intensity blue light was selected to provide sustained activation of the PHOT and CRY receptors, whilst minimising the pronounced morphological and biomass effects, which are associated with high-intensity blue light. In this work, GA- and ABA-deficient mutants, as well as those lacking combinations of *GA2ox* genes and a *GA20ox*-over-expression line, were compared in their response to progressive drought and the different light conditions.

## Materials and methods

### Plant material, growth conditions and sampling

*Arabidopsis thaliana* (L.) Heynh. ecotype Columbia was used as the wild type and all lines were in the Col-0 background. The following mutant and transgenic lines were used: ABA-deficient mutant *aba2* (NASC ID: N3834) (Schwartz et al. 1997), GA-biosynthesis mutants *ga1-3* (transferred from Landsberg *erecta* to Col-0 by six rounds of crossing; Tyler et al. 2004), *ga20ox1/2* (Rieu et al. 2008b), *ga2ox1/2/3/4/6* (Rieu et al. 2008a), *ga2ox7/8* (from lines SALK_055721C and WiscDsLox263B11) (Alonso et al. 2003), and *ga2ox9/10* (from SALK_030540 and SALK_053952C), both mutants kindly provided by Ohad Roth, over-expression line *35S::GA20ox1* (Coles et al. 1999). Seeds were stratified at 4 °C in the dark for 3 d; *ga1-3* was stratified in 5 µM GA_4_ to ensure germination, after which the seeds were sterilised in 1.4% bleach and washed 5 times with sterilised distilled water. Then the seeds were sown in pots measuring 4×4x5.5 cm containing 19 g of sifted soil (Substrate TS 3 medium basic + clay (404) standard, Klasmann-Dielmann Silute, Lithuania). Plants were grown for 2 weeks in long days (LD) (16 h at 23 °C, 8 h dark at 18 °C, then switched to short days (SD) for 3 weeks (8 h light at 23 °C, 16 h at 18 °C), in both cases with 50% relative humidity and light flux of 110 μmol m^− 2^ s^− 1^ in controlled conditions [growth chamber KK 1200 SMART PRO (Nejlabo)]. After 5 weeks, watering was discontinued for the drought-stressed group, whilst the control group was continued to be watered each day to the maximum capacity according to the starting weight. Plants in white light were grown in SD [a full-spectrum LED light source containing approx. 16% blue light (400–500 nm)], as described above, whilst plants in blue light were grown in continuous light with a flux of 10 μmol m^− 2^ s^−1^ at 23 °C, in controlled conditions [growth chamber Microclima 100E (Snijders Scientific, Tilburg, the Netherlands)]. Water loss for the droughty plants was determined by weighing the pots daily. Plants (8 biological replicates per treatment) were harvested after 7 days of drought stress, 6 weeks after germination. Shoots and roots were harvested separately, soil was gently removed by hand from the roots, which were washed briefly with distilled water to remove remaining soil particles and gently dried with paper tissues to remove excess moisture. The shoots and roots were then weighed and frozen in liquid nitrogen, homogenised and stored at − 80 °C for further analysis. To determine leaf areas, plants were photographed and the areas calculated using ImageJ (FIJI) by reference to a grid using a plug-in MACRO (script in Supplementary file S1).

### Evaluation of stress

Relative water content was determined on detached leaves as described in Jiang et al. (2009). Leaf temperature was estimated from thermal images of whole rosettes captured by a thermal camera (IR camera Xi 400, Optris, Berlin, Germany) using Process Imager Software Optris PIX Connect. For each line, 5 plants were scanned for each treatment, and 10 temperature values (averages from a 5×5 pixels area) were recorded for each plant (*n* = 50).

### Hormone analysis

Gibberellin concentrations were determined using an ultra-high-performance liquid chromatograph Acquity UHPLC® I-Class (Waters, Milford, CT, USA) coupled to a tandem mass spectrometer Xevo TQ-S (Waters, Manchester, UK) by modifying a previously published method (Urbanová et al. 2013). Plant material (40 mg fresh tissue) was extracted overnight at 4 °C in 80% aqueous acetonitrile with 5% formic acid containing 3 pmol ^2^H_2_-labelled GA internal standards (Olchemim s.r.o., Olomouc, Czech Republic). After evaporation to dryness, extracts were re-dissolved in 100% methanol (0.3 mL) to which was added 2.7 mL 25 mM NH_4_HCO_3_. The samples were loaded onto activated and equilibrated 30 mg MAX cartridges (Waters), washed with 100% acetonitrile and eluted with 0.2 M formic acid in acetonitrile. After taking the samples to dryness, they were re-dissolved in 10 mM formic acid in methanol (30 µL) from which 3 µL was injected onto the chromatographic column. The mass spectrometer was operated in negative ion and multiple-reaction monitoring mode and the data were processed via MassLynx v4.2 software (Waters). GA concentrations were determined by isotope dilution and expressed as pmol g⁻^1^ fresh weight.

For global hormone analysis, samples were analysed by UHPLC-MS/MS according to a previously published protocol (Šimura et al. 2018). Briefly, 15 mg of fresh plant tissue was extracted in aqueous 60% (v/v) acetonitrile in the presence of a mixture of isotopically labelled internal standards. Homogenization was performed using an Retsch MM 400 oscillation mill (Retsch, Haan, Germany), followed by sonication and centrifugation at 36,000 *g* in a benchtop centrifuge Alegra 64R (Beckman Coulter, Brea, CA, USA). All steps were carried out at approximately 4 °C. After centrifugation, the supernatant was purified using an Oasis® HLB 30 mg/1 cc extraction cartridge (Waters). The flow-through fraction was evaporated to dryness, dried and re-dissolved in aqueous 25% (v/v) acetonitrile (40 µL) from which 5 µL was injected onto the Acquity UPLC® CSH™ C18 column (1.7 µm, 2.1 × 150 mm). Data were acquired in multiple-reaction monitoring mode, processed using MassLynx v4.2 software (Waters), and hormone concentrations were calculated by isotope dilution.

### RNA sequencing and analysis of differential gene expression

Total RNA was isolated from three replicate root (30 mg) and shoot (80 mg) samples using the ISOLATE II RNA Plant Kit (Bioline-Meridan) and treated with Recombinant DNase I (RNase-free) (Top-Bio a.s.) according to the manufacturers protocol. RNA quantity and integrity were measured with an Agilent 2100 Bioanalyzer (Agilent Technologies). RNA sequencing (RNA-seq) was performed on samples with RNA integrity number (RIN) above 6.3 at Novogene Europe (Cambridge, UK) on the Illumina NovaSeq 6000 platform with a paired-end 150 bp sequencing strategy. Raw reads were processed with Trimmomatic v0.39 (Bolger et al. 2014) to remove Illumina adapter sequences, bases with a Q-score below 13, and reads shorter than 36 bp. High-quality clean reads were aligned to the Arabidopsis reference genome using HISAT2 v2.1.0 (Kim et al. 2019) with default parameters. Gene-level read counts were obtained with the feature Counts function in the Rsubread v2.0.1 package (Liao et al. 2019), using only uniquely mapped reads. Differential expression analysis was carried out with DESeq2 (Love et al. 2014). Log_2_-fold change (LFC) values were shrunk using the lfcShrink function with the ASHR method. Genes were considered differentially expressed if they had an FDR-adjusted *P*-value < 0.05 (Benjamini–Hochberg correction) and an absolute LFC ≥ 1 (≥ 2-fold change). For downstream analyses, count data were transformed with the regularised logarithm (rlog). Hierarchical clustering (HCA) and principal component analysis (PCA) were then performed. Heatmaps were generated using the ComplexHeatmap v2.2.0 R package (Gu et al. 2016).

### Gene expression by RT-qPCR

Total RNA was extracted from at least 3 biological replicates (in some cases from pooled samples to reach sufficient weight for a robust analysis) using ISOLATE II RNA Plant Kit (Bioline-Meridan) and treated with Recombinant DNase I (RNase-free) (Top-Bio a.s.). cDNAs were synthesised by PrimeScript™ 1 st strand cDNA KIT (TaKaRa) using Ribonuclease H (TaKaRa). The RT-qPCR analysis was performed in technical triplicates using the CFX96 Real-Time System with a C1000 thermal cycler (Bio-Rad). The reaction mixture contained 500 nM primers and SensiFAST SYBR Lo-ROX Kit (Bioline) in a total volume of 12 µL. Efficiencies of primer pairs used for PCR were determined using dilution series of cDNA and standard curve method. *PP2A* (AT1G13320) was used as the reference gene. The primer sequences are listed in Supplementary file 5 Table S1. Relative gene expression was calculated with respect to primer amplification efficiency and the reference gene as described in Hellemans et al. (2007).

### Gene ontology (GO) enrichment analysis

Gene Ontology (GO) enrichment analysis was conducted using over-representation analysis (ORA) with the g:Profiler tool, accessed through the gprofiler2 R package v0.2.3 (Kohlberg et al. 2020). Differentially expressed genes were selected based on an adjusted *P*-value (FDR < 0.05) and a log₂ fold change threshold of ≥ 1 for up-regulated genes or ≤ −1 for down-regulated genes. Up- and down-regulated gene sets were analysed separately. Gene identifiers were converted to Arabidopsis gene names using g:Profiler’s identifier conversion utility, excluding unmapped genes. GO enrichment was evaluated using g:Profiler’s statistical framework with multiple testing correction, and significantly enriched GO terms were summarised using gene ratio and background ratio metrics.

### Multivariate analysis

Multivariate analysis of growth parameters and hormone concentrations was performed in R v4.3.0 (R Core Team, Vienna, Austria) using RStudio 2023.06.0 Build 421 (Posit Software, PBC, Boston, MA, USA) with packages: readxl, MuMIn, ggplot2 and ggpubr. Morphological traits, including leaf area, leaf fresh weight, and root fresh weight, were merged with plant hormone data. Missing values representing concentrations below the quantification limit were replaced by two-thirds of the minimum non-missing value within the respective variable. Irrigation and light treatments were coded as −1/1 contrasts, with irrigated = −1 and drought = 1, and white light = −1 and blue light = 1. The dataset was split by organ into shoot and root subsets. Within each subset, all response variables were mean-centred and scaled to unit standard deviation. For each response, the optimal linear model describing the effects of drought, blue light, and their interaction was selected from all relevant candidate models using the corrected Akaike information criterion (AICc; Hurvich and Tsai 1989). The model with the lowest AICc was retained. Estimated model coefficients were visualised as effects in a blue–white heatmap, where blue indicates negative effects, red indicates positive effects, and white indicates effects close to zero. Coefficient significance was assessed using t-statistics and expressed as *P*-values represented by asterisks. Effects excluded during AICc model selection were set to zero before visualisation.

## Results

### Effect of water restriction on seedling growth and hormone content under white and blue light

**Fig. 1:**
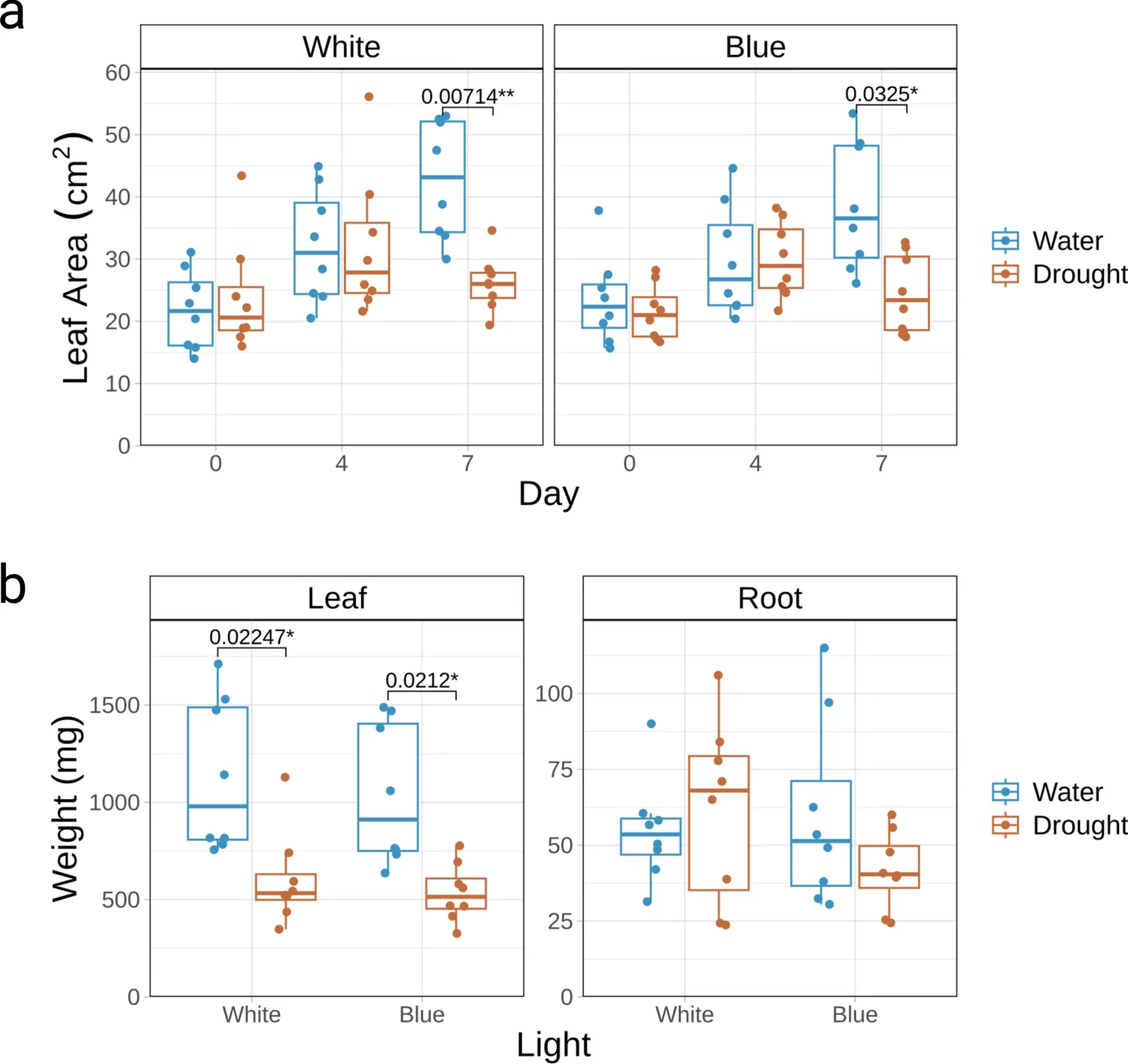
Growth parameters of 5-week-old Arabidopsis plants under water restriction, grown in short-day white light (8 h light at 23 °C, 16 h at 18 °C) and continuous blue light (14 h light at 23 °C), in 8 biological replicates per treatment. **a** Rosette areas were measured in watered and drought-stressed conditions, 0, 4 and 7 days after watering was discontinued. **b** Shoot and root weights after 7 days. Differences between watering treatments were tested using two-sample *t*-tests with Holm correction for multiple comparisons. Exact adjusted *P*-values are shown for all comparisons, with significance indicated by asterisks (*P ≤ 0.05, **P ≤ 0.01)

Arabidopsis Columbia (Col-0) seedlings were grown for 2 weeks in long days (LD) and then in short days (SD) for 3 weeks to delay flowering, after which plants were transferred to different light regimes. Watering was stopped for half the plants in each light regime, whilst the other half continued to be watered to 100% capacity. The light conditions were SD white light or continuous blue light. After 7 days the soil water content after water restriction in white and blue light was reduced to 21 and 24%, respectively. The effect of drought on growth parameters is presented in Fig. 1. Leaf areas were reduced in drought-stressed plants by 39 and 36%, respectively (Fig. 1a), whilst shoot fresh weights were reduced by 46 and 48%, respectively, compared to watered plants in white and blue light (Fig. 1b). There was no statistically significant difference in root weight between watered and stressed plants under either light condition (Fig. 1b).

**Fig. 2:**
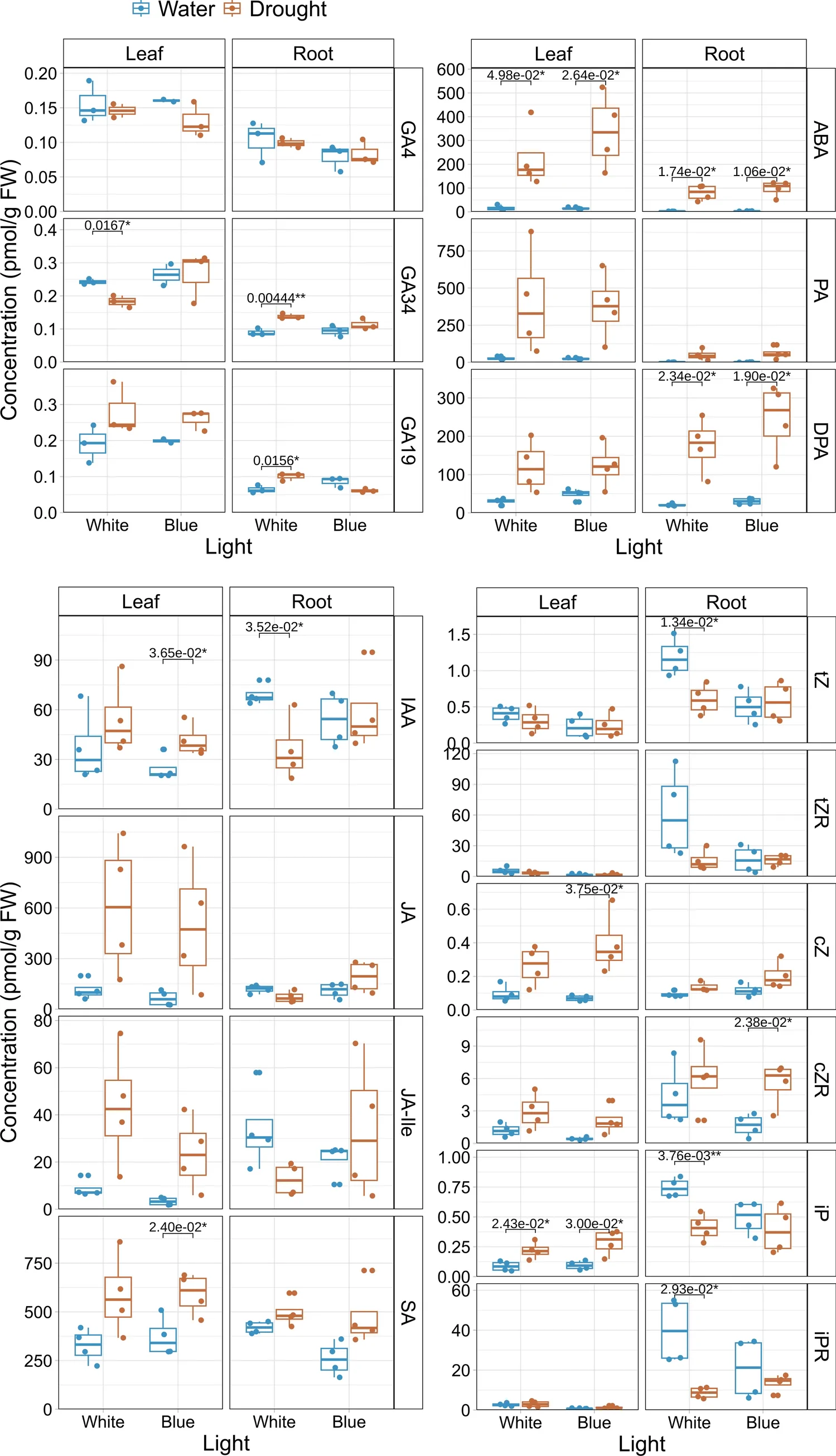
Concentrations of selected hormones in watered and drought-stressed Arabidopsis plants. Water restriction was applied to 5-week-old Arabidopsis plants and the hormones analysed after 7 days. GAx, gibberellin Ax; *c*Z, *cis*-zeatin; *c*ZR, *cis*-zeatin riboside; DPA, dihydrophaseic acid; iP, isopentenyladenine; iPR, isopentenyladenine riboside; *t*Z, trans-zeatin; *t*ZR, trans-zeatin riboside; ABA, abscisic acid; IAA, indoleacetic acid; JA, jasmonic acid; JA-Ile jasmonic acid isoleucine conjugate; PA, phaseic acid; SA, salicylic acid. Differences between watering treatments were tested using two-sample *t*-tests with Holm correction for multiple comparisons. Exact adjusted *P*-values are shown for all comparisons, with significance indicated by asterisks (*P ≤ 0.05, **P ≤ 0.01)

Hormone contents were determined in the shoots and roots of watered and stressed plants (Fig. 2, Supplementary files 6 and 7 Tables S2 and S3). For technical reasons, GA content was measured in a separate experiment from the other hormones. The concentration of GA_4_ did not change significantly in leaves or roots due to drought under both light conditions (Fig. 2). However, the concentration of its 2β-hydroxy catabolite GA_34_ was significantly reduced by drought in shoots and increased in roots under white light, but did not change significantly in blue light (Fig. 2, Supplementary file 6 Table S2). In addition, the 13-hydroxy intermediate GA_19_ increased slightly under drought in shoots under both light conditions, whilst in roots, it increased in white light but decreased in blue light.

ABA metabolites were analysed as part of a global hormone analysis, together with auxins and precursors, cytokinins, jasmonates and precursors, and salicylic acid (SA). Their concentrations are presented in Supplementary file 7 Table S3, and for the main hormones in Fig. 2. Drought stress significantly increased ABA levels in both organs and light conditions, with higher overall concentrations in shoots, although there was a greater increase in roots, approximately 38-fold under both light conditions, as opposed to 14–23-fold in leaves. Concentrations of the ABA catabolites phaseic acid (PA) and dihydrophaseic acid (DPA) were elevated by drought in both organs and light conditions although only the increase of DPA in roots had high statistical significance. IAA concentrations in shoots increased under drought under both light conditions, whereas in roots, drought had little effect on IAA under blue light, but caused a decrease under white light. Tryptophan, a precursor of IAA, increased substantially under drought in both organs: approximately 6-fold in roots, and 15-fold in leaves under white and blue light. IAN, another IAA precursor, also increased in both roots and leaves, particularly under blue light. Cytokinin profiles showed elevated levels of *cis*-zeatin (*c*Z), its riboside (*c*ZR), and related conjugates (*c*ZOG, *c*ZROG, *c*Z7G, *c*Z9G) in leaves under drought in both light conditions, with smaller changes in roots. There were generally small changes in the levels of *trans*-zeatin (*t*Z) and its riboside due to drought, except for a significant reduction in tZ concentration in roots under white light. The levels of *t*Z conjugates (*t*ZOG, *t*ZROG, *t*Z7G, *t*Z9G) were increased by drought with a highly significant increase in tZOG in leaves under blue light. The concentrations of isopentenyladenine (iP) and its riboside (iPR) and glucosides (iP7G, iP9G) decreased in roots under drought but increased in leaves. Jasmonic acid (JA), its conjugate JA-Ile, and biosynthetic precursors *cis*−12-oxo-phytodienoic acid (*cis*-OPDA) and dinor-oxo-phytodienoic acid (dn-OPDA) increased in stressed leaves under both light conditions. Additionally, 12-OH-JA, a JA deactivation metabolite, showed a significant increase in drought-stressed leaves under blue light. Salicylic acid levels were elevated by drought in shoots and roots, but only the increase in blue light was statistically significant.

The effects of drought, blue light, and their interaction on growth parameters and hormone metabolite concentrations were further investigated by multivariate analysis and are displayed as heatmaps in Supplementary file 4 Fig. S1, with separate maps for GAs (Fig. S1a) and other hormones (Fig. S1b) as the data were obtained from different experiments. Only statistically significant effects are shown. Overall, a greater number of significant interactions were observed in roots. There was no interaction between drought and blue light in the promotion of GA_34_ concentration in roots, whereas induction of GA_19_ level by blue light was promoted in roots under drought. Due to high variance in GA_34_ concentration in leaves under blue light, the drought-induced reduction in white light is lost in the multivariate analysis. Induction of ABA and its metabolites by drought was not affected by light conditions in roots or shoots. In roots, drought and blue light synergistically repressed the levels of *t*Z and *t*ZR. Whilst drought and blue light independently promoted cZ content, blue light-induction of its metabolite *c*Z7G was enhanced by drought, as were the levels of JA and its precursors *cis*-OPDA, dn-OPDA. Furthermore, drought-induced accumulation of 12-OH-JA in shoots was promoted in blue light.

### Effect of water restriction on gene expression under white and blue light

Global transcript abundance was determined by RNA sequencing (RNA-seq) in shoots and roots of watered and drought-stressed plants growing in SD white light and continuous blue light. The full dataset of expressed genes showing their differential expression in response to water status in shoots and roots under white or blue light is presented in Supplementary file S2. Principal component analysis (PCA) shows that the largest discrimination is between plant organs, followed by water/drought and then light condition (Supplementary file 4 Fig. S2a). Drought increased the number of differentially expressed genes (DEGs) between white and blue light, with 652 and 204 DEGs (log_2_-fold change <− 1 or > 1) in shoots and roots, respectively, in watered plants, whilst the numbers were 886 and 1955, respectively, for stressed plants (Supplementary file 4 Fig. S2b). Blue light conditions reduced the number of DEGs in response to drought with 6012 in shoots under white light and 3415 in blue light. The equivalent values for roots were 6393 and 3971, respectively. This suggests that plants growing in blue light experienced less drought stress. Indeed, the stress-induced gene *RD29A* was up-regulated 17.6-fold by drought in shoots in white light, but only 3.4-fold in blue light (File S2). The equivalent values for roots were 169.4-fold and 34.5-fold, respectively.

**Fig. 3:**
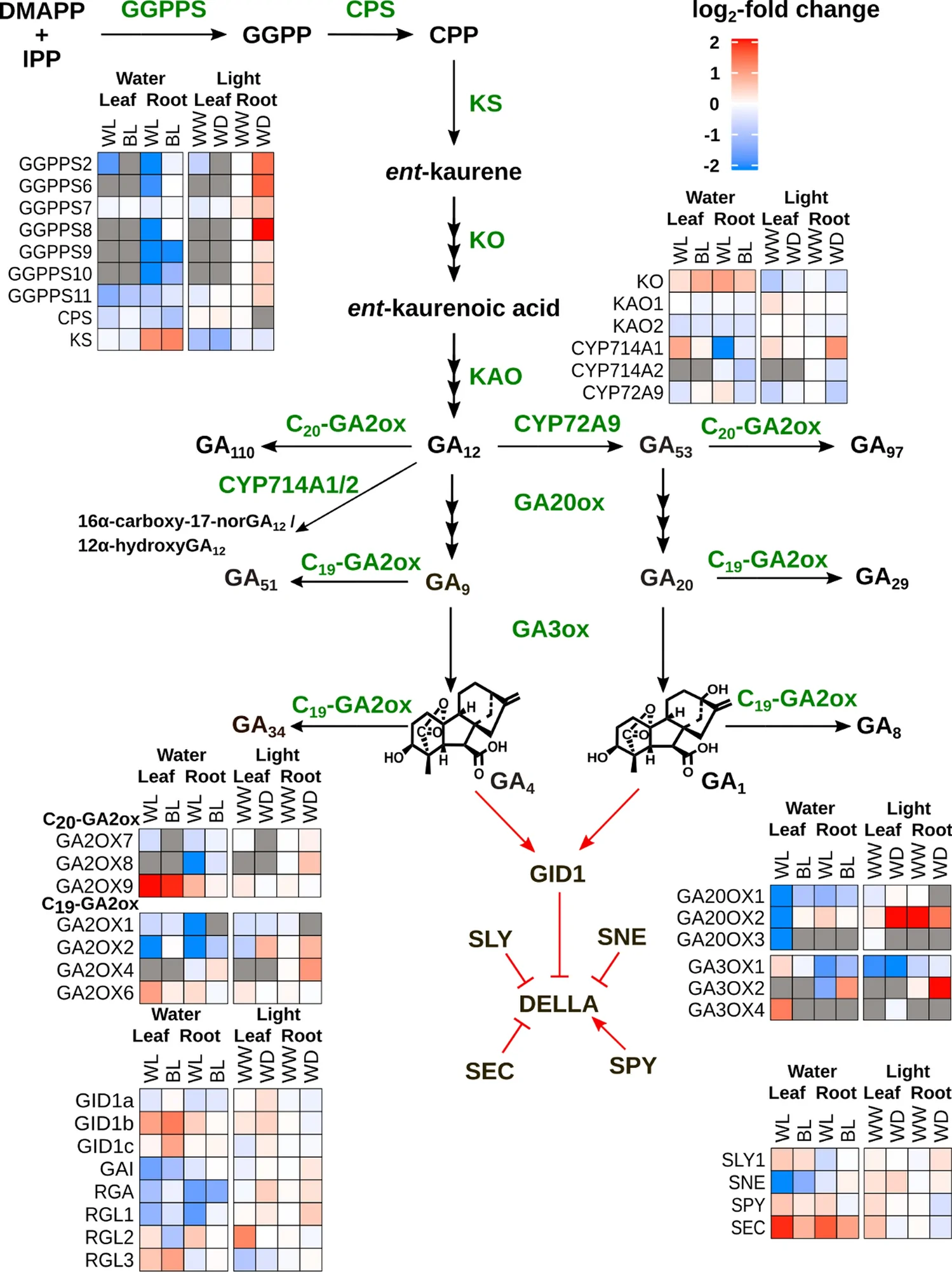
Heat maps for differential expression of genes for GA metabolism and signalling in leaves and roots of watered and drought-stressed Arabidopsis plants growing in short-day white light or continuous blue light. Grey boxes indicate that the number of reads are < 10. Water, differential expression between watered and water-restricted plants: *WL* white light, *BL* blue light. Light, differential expression between white and blue light: WW, watered; WD, drought-stressed

**Fig. 4:**
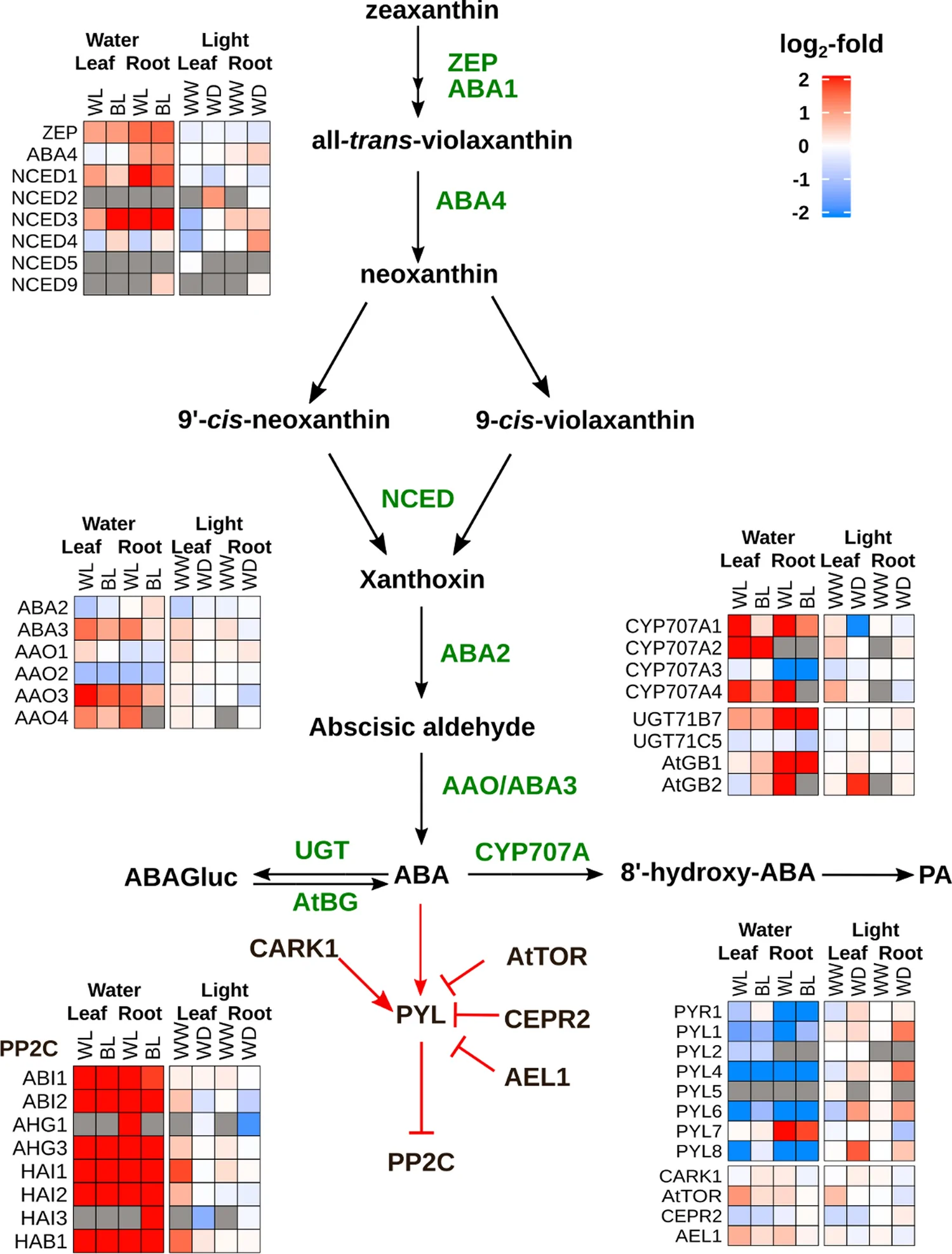
Heat maps for differential expression of genes for ABA metabolism and signalling in leaves or roots of watered and drought-stressed Arabidopsis seedlings growing in short-day white light or continuous blue light. Grey boxes indicate that the number of reads are < 10. Water, differential expression between watered and water-restricted plants: *WL* white light, *BL* blue light. Light, differential expression between white and blue light: WW, watered; WD, drought-stressed

Results for genes involved in the metabolism and signalling for GAs and ABA are listed in Supplementary file S3 which shows the numbers of normalised reads and differential expression as log_2_-fold changes in response to water and light conditions. Highly significant changes (*P* < 0.05) are in bold type whilst changes > 2-fold (log_2_ = 1) are highlighted. The data are presented as heatmaps for GA and ABA (Figs. 3, 4). It is unclear which *GGPPS* genes are responsible for GA biosynthesis, but of the plastid-localised enzymes, only *GGPPS2*,* 7* and *11* were expressed in both shoots and roots. *GGPPS2 and 11* were down-regulated by drought in shoots in white light but much less so in blue light. All seven *GGPPS* genes were expressed in roots with five genes *GGPPS2,6, 8, 9* and *10* strongly down-regulated by drought in white light. However, only *GGPPS9* and 10 were also down-regulated by drought in blue light and less strongly than in white light. Of the early GA-biosynthetic genes, only *ent*-kaurene synthase (*KS*) showed sensitivity to the experimental conditions. Its expression was up-regulated by drought in roots under both light conditions. There was little change in response to drought in shoots, in which it was down-regulated by blue light. Expression of both *AtGA20ox1* and* 2* was reduced by drought under white light in shoots and for *AtGA20ox1* also in roots. Down-regulation of *AtGA20ox1* by drought was much reduced in blue light, whilst expression of *AtGA20ox2* was strongly up-regulated under blue light in drought-stressed shoots and in roots in both watered and stressed plants. This may have masked effects of drought on its expression under these conditions.

Expression of *AtGA3ox1*, the most highly expressed *GA3ox* gene, was unaffected by drought in shoots, but decreased in roots under both light conditions. *AtGA3ox2* was expressed at very low levels in shoots, whilst in roots it was down-regulated by drought in white light, but up-regulated in blue light, which strongly promoted its expression under drought but not in watered roots. The expression of most *GA2ox* genes was influenced by drought in shoots and/or roots. In shoots, the most highly expressed genes were *GA2ox1*, *GA2ox2* and *GA2ox6.* Under white light, the expression of *GA2ox1* was reduced slightly by drought and there was strong down-regulation of *GA2ox2*, whilst *GA2ox6* expression was up-regulated (log_2_-fold induction 0.98, *P* = 0.037). These changes did not occur in blue light. The C_20_-GA2ox gene *GA2ox9*, was strongly up-regulated by drought under both light conditions. The most highly expressed *GA2ox* genes in roots were *GA2ox2*, *GA2ox6* and *GA2ox9*. *GA2ox2* was down-regulated by drought, particularly in white light, whilst there was a small, but significant up-regulation of *GA2ox9* only in white light, but no significant change in *GA2ox6* expression. In white light, roots contained higher levels of *GA2ox7* and *GA2ox8* transcripts than shoots, and notably the expression of *GA2ox8* was strongly down-regulated by drought, but only in white light.

The expression of *CYP14A1*, which encodes an inactivating monooxygenase (Nomura et al. 2013), was reduced by drought in roots under white light, but much less in blue light. Expression of the GA receptor gene *GID1B* was up-regulated by drought in leaves, but not in roots, under both light conditions, whilst *GID1C* was up-regulated by drought only in blue light. Expression of the genes encoding DELLA proteins was sensitive to water status. *GAI* expression was down-regulated by drought in leaves under both light conditions, whilst *RGA* was down-regulated in leaves in white light and in roots under both light conditions. Expression of *RGL1* was also down-regulated by drought in leaves and roots under white light, whilst *RGL2* expression was down-regulated in leaves under blue light. *RGL3* was the only DELLA gene whose expression was promoted by drought, and only in leaves. Expression of the F-box-encoding gene *SNEEZY* (*SNY*) was reduced in leaves under drought in both light conditions, whilst *SECRET AGENT* (*SEC*), which encodes an *N*-acetylglucosamine transferase that modifies DELLA function (Zentella et al. 2016), was strongly up-regulated by drought in leaves and roots, particularly in white light.

There was strong regulation of ABA metabolism and signalling genes in response to drought (Fig. 4) with a slightly larger effect under white light than under blue light. The expression of the biosynthetic genes *ZEP* (*ABA1*), encoding ZEAXANTHIN EPOXIDASE, *ABA4*, required for neoxanthin synthesis, and *NCED1*, *2* and *3*, encoding 9-*cis*-epoxycarotenoid dioxygenases, was up-regulated by drought in roots more highly than in leaves, with little effect of light condition. However, *NCED4*, the most highly expressed *NCED* gene in shoots, was slightly down-regulated by drought in both shoots and roots under white light, with little differential expression in blue light. The most highly regulated gene *NCED3* showed high up-regulation in drought conditions with a smaller increase in leaves under white light. The *CYP707A* genes, which encode ABA 8’-hydroxylase, were up-regulated by drought in shoots and roots under both light conditions. The only exception in this group was *CYP707A3,* which was unaffected in shoots and down-regulated in roots. Genes encoding the PP2C protein phosphatases, involved in ABA signal transduction, were universally up-regulated by drought in leaves and roots under both light conditions, whilst expression of the *PYR/PYL/RCAR* ABA-receptor genes was reduced under drought. The exception is *PYL7*, which was up-regulated by drought in roots under both light conditions.

Gene ontology (GO) over-representation analysis (ORA) of differentially expressed genes across tissues and conditions in response to drought is shown for biological processes and KEGG pathways (Supplementary file 4 Fig. S3a and b). Cellular processes are strongly represented by down-regulated genes in shoots, whilst response to stimulus and metabolism, including biosynthesis of secondary metabolites are represented by both up- and down-regulated genes in both shoots and roots under both light conditions. Response to stress and to ABA are up-regulated processes.

### Comparison of gene expression in Col-0 with ABA- and GA-deficient mutants

**Fig. 5:**
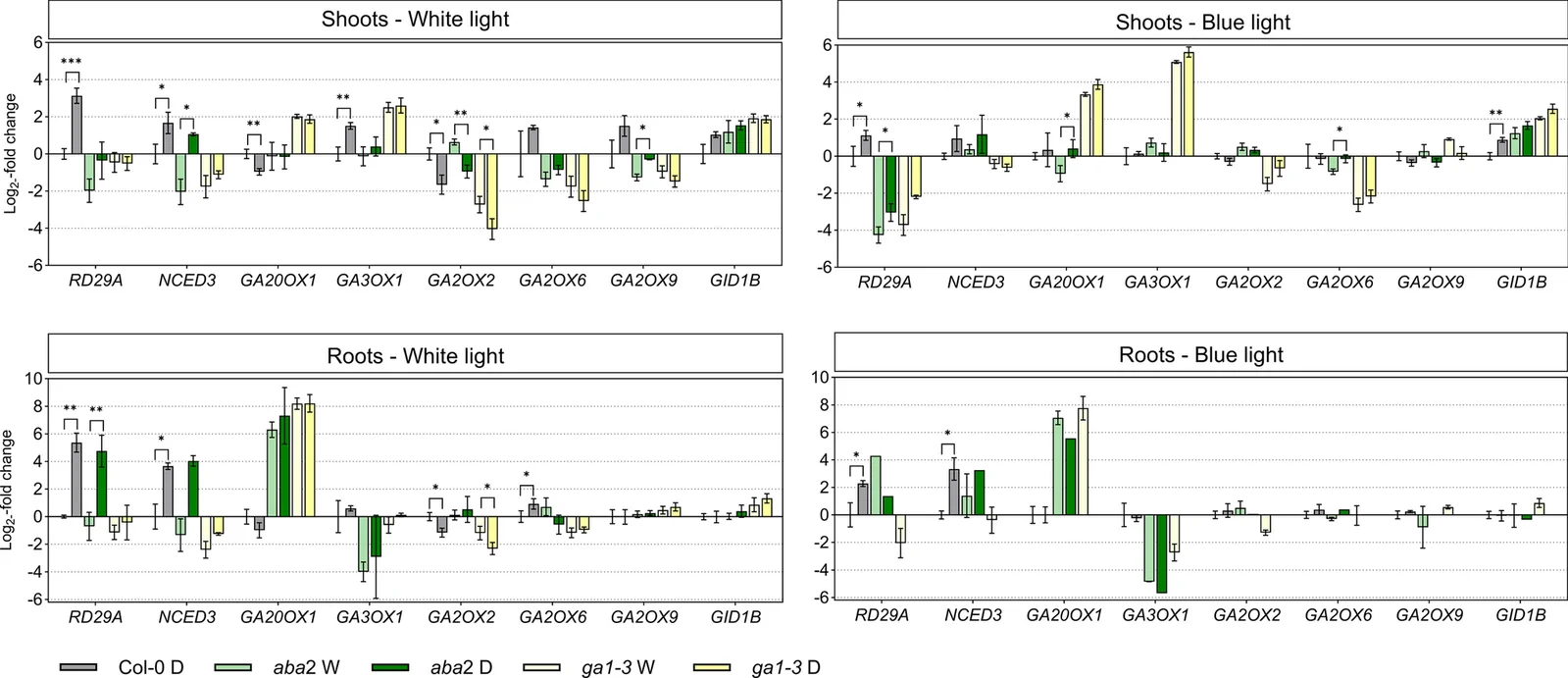
Relative expression of selected genes in leaves (shoots) and roots of Col-0, ABA-deficient (*aba2*) and GA-deficient *(ga1-3*) mutants growing in short-day white and continuous blue light under watered and water-restricted conditions. Transcript levels determined by RT-qPCR are plotted relative to watered Col-0 separately for white and blue light. There are no values for drought-stressed *ga1-3* roots in blue light as there was insufficient material. The effect of drought was evaluated separately for each gene and genotype by comparing water and drought treatments using two-sided Welch’s *t*-tests. Only statistically significant differences are indicated by asterisks (*P ≤ 0.05, **P ≤ 0.01, ***P ≤ 0.001)

The changes in gene expression in drought-stressed Col-0 seedlings in white and blue light were verified by RT-qPCR for a selection of GA metabolism genes, the ABA biosynthesis gene *NCED3* and the stress gene *RD29A* (Fig. 5). In this experiment, Col-0 was compared with the GA- and ABA-deficient mutants *ga1-3* and *aba2*, respectively*.* Rosette and root weights as well as rosette areas are shown in Supplementary file 4 Fig. S4 for watered and stressed plants 7 days after watering was discontinued. As noted previously, growth in blue light was less affected by drought compared with plants growing in white light. Growth of the *aba2* and *ga1-3* lines was highly restricted under all water and light conditions with no effect of water restriction on shoot growth, although there was a reduction in root weight in the mutants particularly in blue light from which there was insufficient root material from *ga1-3* for RT-qPCR analysis. Figure 5 shows the difference in gene expression relative to watered Col-0 whilst gene expression calculated as log_2_(-ΔCt) values relative to the reference gene (PP2A) are presented in Supplementary file 4 Fig. S5 to visualise differences between genotypes and light conditions. The RT-qPCR values for Col-0 largely confirm the RNA-seq data with water restriction resulting in increased expression of *RD29A* and *NCED3*, more strongly in roots compared to shoots and less in blue compared to white light. Under drought in white light, there was decreased expression of *AtGA20ox1* and *AtGA20ox2* and increased expression of *AtGA3ox1, AtGA2ox6, AtGA2ox9* and the receptor gene *AtGID1B* in shoots with a similar trend in roots except there was little change in the expression of *AtGA2ox9*, which is expressed more highly in roots than shoots, and *AtGID1B.* In blue light, these changes in gene expression in response to drought were much reduced or absent.

The differences in expression of *RD29A* and *NCED3* in *aba2* relative to Col-0 under white light followed a similar trend. Expression of *RD29A* in *aba2* shoots was much lower than in Col-0 under both light conditions, with smaller increases in drought-stressed shoots, consistent with the response of *RD29A* expression to drought being partially mediated by ABA signalling (Narusaka et al. 2003). However, in roots there was a much larger increase in expression under drought in white light indicating an ABA-independent response. In white light, the expression of *NCED3* in watered *aba2* shoots and roots is lower than in Col-0, but with strong drought-associated up-regulation, whilst in blue light drought had a smaller effect on expression. In *aba2* roots, but not shoots, *AtGA20ox1* expression was substantially higher than in Col-0 under both light conditions and, in white light, in stressed as well as watered plants. This is consistent with the down-regulation of *AtGA20ox1* expression by ABA signalling reported for Arabidopsis stems (Son et al. 2010) although this effect was not present in rosettes. The increased *AtGA20ox1* expression in *aba2* roots was accompanied by decreased *AtGA3ox1* expression, also observed in stems by Son et al. (2010), potentially through the homeostatic mechanism. Reduced expression of *AtGA2ox6* in *aba2* shoots from watered plants relative to Col-0 in both light conditions, as noted also for *RD29A* and *NCED3*, indicates that these plants experience less stress than watered Col-0. This is supported by higher expression of *AtGA2ox2*, which is down-regulated by drought stress in Col-0. Under water restriction, *AtGA2ox2* expression is reduced in shoots, as in Col-0, whilst expression of *AtGA2ox6* is less strongly up-regulated than in Col-0. In roots, the difference in expression of the *ga2ox* genes relative to Col-0 in watered plants is less clear with drought producing slight up-regulation of *AtGA2ox2* and down-regulation of *AtGA2ox6.* As in Col-0, expression of *AtGA2ox9* was unaffected by drought in roots. The expression of *GID1B* was higher in *aba2* shoots relative to Col-0 under both light conditions with little change due to drought, whilst expression levels in roots were similar to those in Col-0.

*RD29A* and *NCED3* expressions in *ga1-3* were lower than in Col-0 irrespective of organ, water or light condition, suggesting that these plants experienced little drought stress under the experimental conditions. *AtGA20ox1* and *AtGA3ox1* were up-regulated in *ga1-3* shoots relative to Col-0 under both water and light conditions, in agreement with feedback suppression of biosynthesis (Thomas et al. 1999). Whilst *AtGA20ox1* was also up-regulated in roots of *ga1-3*, the expression of *AtGA3ox1* was similar to that in Col-0 roots in white light and lower in blue light, indicating tissue-specific differences in homeostasis. The *GA2ox* genes were expressed at relatively low levels in *ga1-3* shoots under both light conditions consistent with reduced expression in response to low GA levels (Thomas et al. 1999), whilst in roots there was little difference in expression from Col-0. *GID1B* expression was up-regulated relative to Col-0 in *ga1-3* shoots and roots under both light conditions consistent with its regulation by GA signalling (Griffiths et al. 2006; Middleton et al. 2012). There was little difference in expression of the GA genes between watered and water-restricted *ga1-3* plants supporting a lack of stress in these plants.

### Drought responses in lines with altered GA metabolism: growth, leaf temperatures and gene expression

**Fig. 6:**
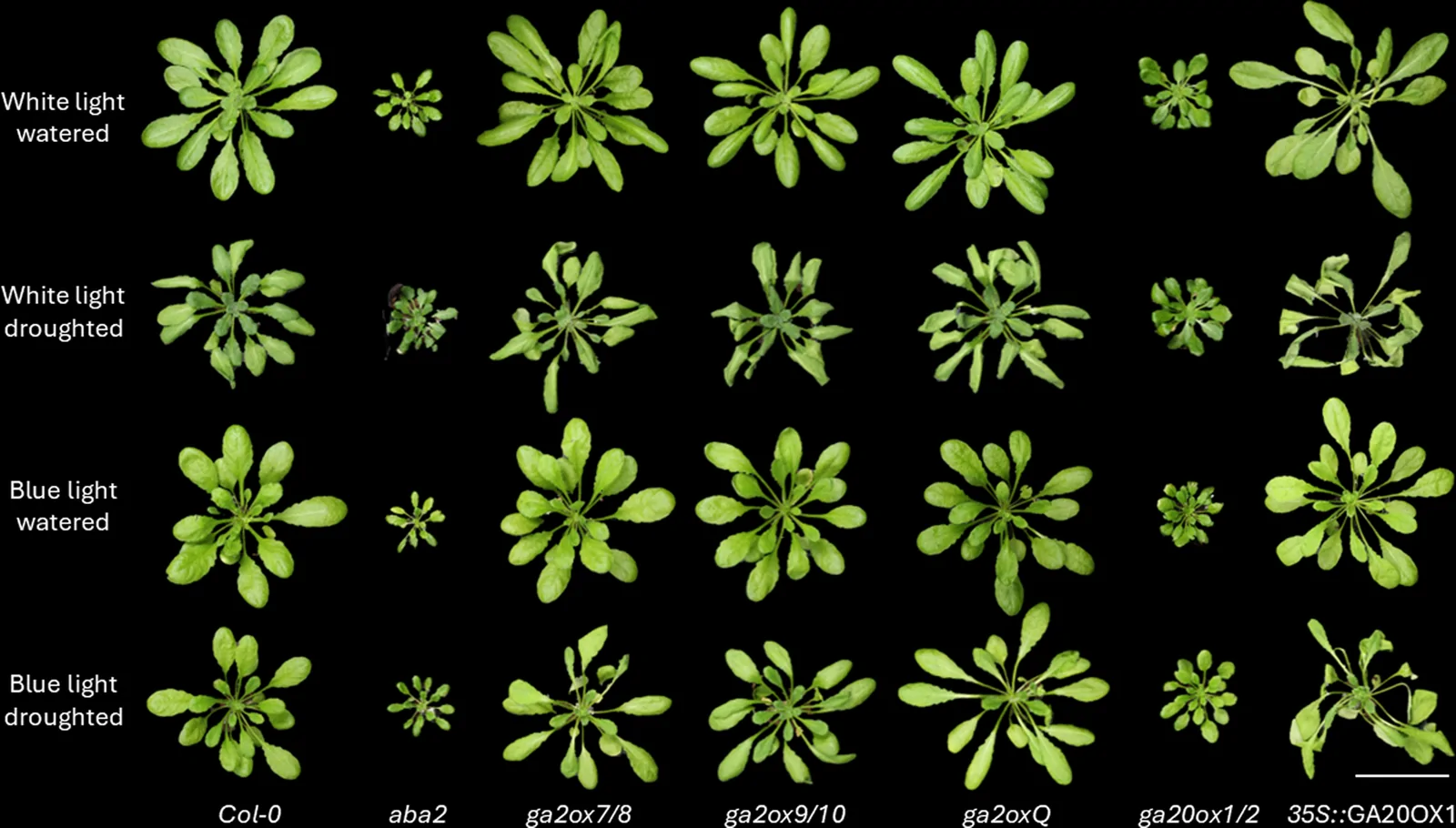
Photographs of representative lines of WT (Col-0), *aba2*, *ga2ox7,8* and *ga2ox9,10* double mutants, *ga2ox1,2,3,4,6* quintuple mutant (*ga2oxQ*), the GA-deficient *ga2ox1,2* double mutant and the *35S::AtGA20ox1* over-expression line growing in short-day white and continuous blue light under watered and drought-stressed conditions for 7 days, scale 5 cm

The changes in expression of GA genes in response to water restriction indicated that Arabidopsis responded to the stress by both reducing GA biosynthesis and increasing GA catabolism. This was investigated further by determining the response to drought and blue light of mutants lacking functional *GA20ox1* and *GA20ox2* (*ga20ox1/2*), the quintuple *ga2ox1,2,3,4,6* mutant (*ga2oxQ*) lacking all C_19_-GA2ox enzymes as well as the double mutants *ga2ox7/8* and *ga2ox9/10* which lack these C_20_-GA2ox sub-clades (Schomburg et al. 2003; Lange et al. 2020). Their response was compared with that of the over-expression line *35S-AtGA20ox1* with high GA content (Coles et al. 1999), *aba2* as well as Col-0. Figure 6 compares the shoots of these lines under the applied water restriction and light conditions.

**Fig. 7:**
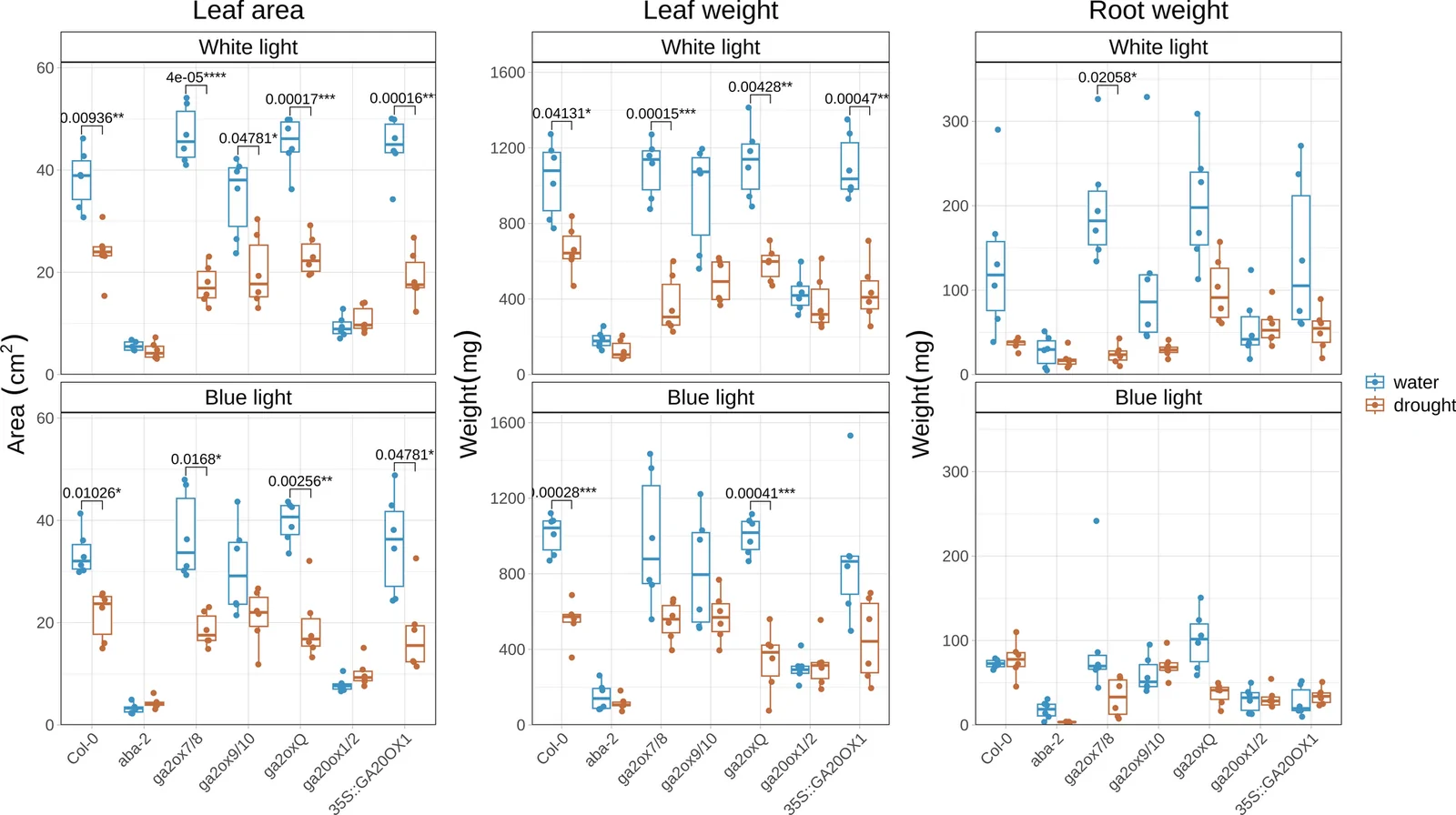
Leaf areas, leaf (shoot) and root weights in plants shown in Fig. 6. WT (Col-0), *aba2*, *ga2ox7,8* and *ga2ox9,10* double mutants, *ga2ox1,2,3,4,6* quintuple mutant (*ga2oxQ*), the GA-deficient *ga2ox1,2* double mutant and the *35S::AtGA20ox1* over-expression line growing in short-day white and continuous blue light under watered and water-restricted conditions. Differences between watering treatments were tested using two-sample *t*-tests with Holm correction for multiple comparisons. Exact adjusted *P*-values are shown for all comparisons, with significance indicated by asterisks (*P ≤ 0.05, **P ≤ 0.01, ***P ≤ 0.001)

The *ga20ox1/2* double mutant was shown previously to be more tolerant of drought than Col-0, whilst the *ga2oxQ* and *35S::AtGA20ox1* lines are at least as sensitive as Col-0 (Colebrook et al. 2014). Under both light conditions, shoot growth determined by rosette area and weight were reduced by water restriction in Col-0, *ga2oxQ, ga2ox7/8*, *ga2ox9/10* and *35S::AtGA20ox1*, but not in *aba2* and *ga20ox1/2* (Fig. 7)*.* Under white light, with the exception of *aba2* and *ga20ox1/2*, root weight was reduced slightly by drought with the largest reduction in *ga2ox7/8*.

Relative to white light, root growth in all lines was lower in blue light in watered plants and reductions under drought were found only in *ga2ox7/8*, *ga2oxQ* and *aba2*. In contrast to the other lines, the *AtGA20ox1* over-expression line was wilted by 7 days water restriction under both light conditions (Fig. 6) as indicated also by the relatively large reduction in relative water content (RWC) under drought compared with most other lines (Supplementary file 4 Fig. S6). The relative water content of *ga2ox7/8* was also substantially reduced by drought under both light conditions indicating an involvement of one or both of these genes in the response to drought.

**Fig. 8:**
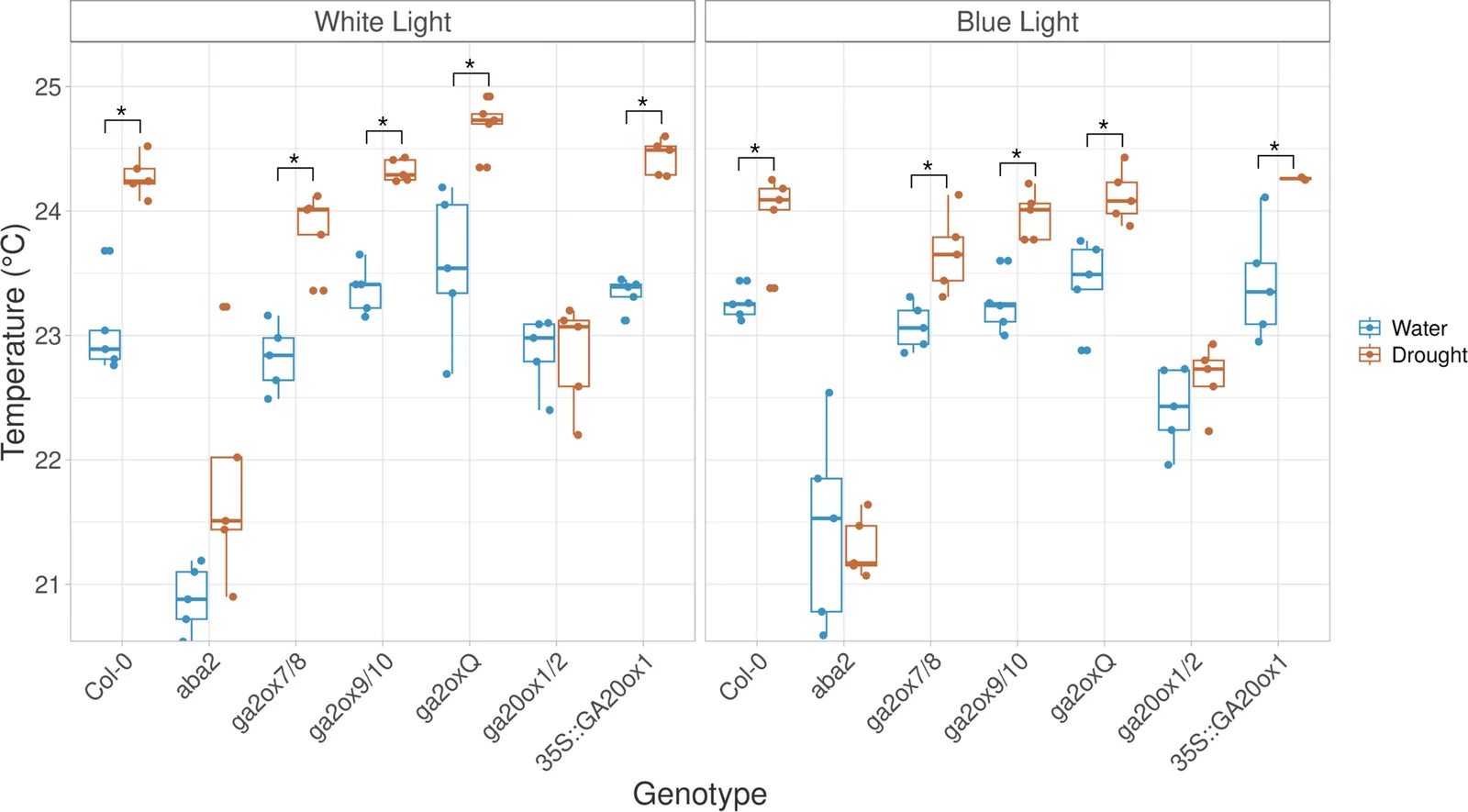
Leaf temperatures measured in the plants as shown in Fig. 6. The temperatures and significance levels (*P*-values) for differences between watered and drought-stressed plants, plants grown in white and blue light, and between Col-0 and the other lines are given in Supplementary file 8 Table S4. Statistically significant differences between watering treatments are indicated (*P ≤ 0.05)

As a proxy for stomatal status, leaf temperatures were compared between the WT, mutant and over-expression lines under watered and drought-stressed conditions in both white and blue light (Fig. 8, Supplementary file 8 Table S4). Overall, differences in leaf temperature between well-watered and drought-stressed plants were smaller under blue light than under white light, with leaf temperature generally lower under blue light across all genotypes, consistent with stomatal closure reducing transpirational cooling. By contrast, the *aba2* mutant maintained lower leaf temperature than Col-0 under both watered and drought-stressed conditions, reflecting the reduced ability to close stomata, although there was a slight increase (*P* = 0.076) in leaf temperature in response to drought in white light. The GA-deficient *ga20ox1/2* mutant showed little or no increase in leaf temperature under drought. Together with the unchanged relative water content (Supplementary file 4 Fig. S6), this suggests that the muted response is more likely a consequence of its reduced plant size and lower water use than impaired stomatal regulation. Under white light, the leaf temperature of *ga20ox1/2* was comparable to that of Col-0 under well-watered conditions, indicating similar transpiration rates, whereas under blue light it remained lower, suggesting light-dependent regulation of transpiration. Overall, these findings indicate that the reduced stomatal closure observed in *ga20ox1/2* is more likely a secondary effect of its lower water demand than a primary defect in stomatal regulation. Col-0 and the other lines showed significant increases in leaf temperature under drought reflecting the stomatal closure. Notably, the leaf temperature of the *ga2ox7/8* mutant was slightly lower than Col-0 and the other *ga2ox* mutants and the over-expression line under all water and light conditions, although its leaf temperature increased under drought as effectively as in the other lines. Water loss from the *aba2* and *ga20ox1/2* mutants was much less than for the other lines under both light conditions. The *ga20ox7/8* mutant and *GA20ox1*-over-expression line lost significantly more water than Col-0 in white light, whilst in blue light the greatest water loss was shown by the *ga2oxQ* mutant (*P* = 0.058) (Supplementary file 4 Fig. S7).

**Fig. 9:**
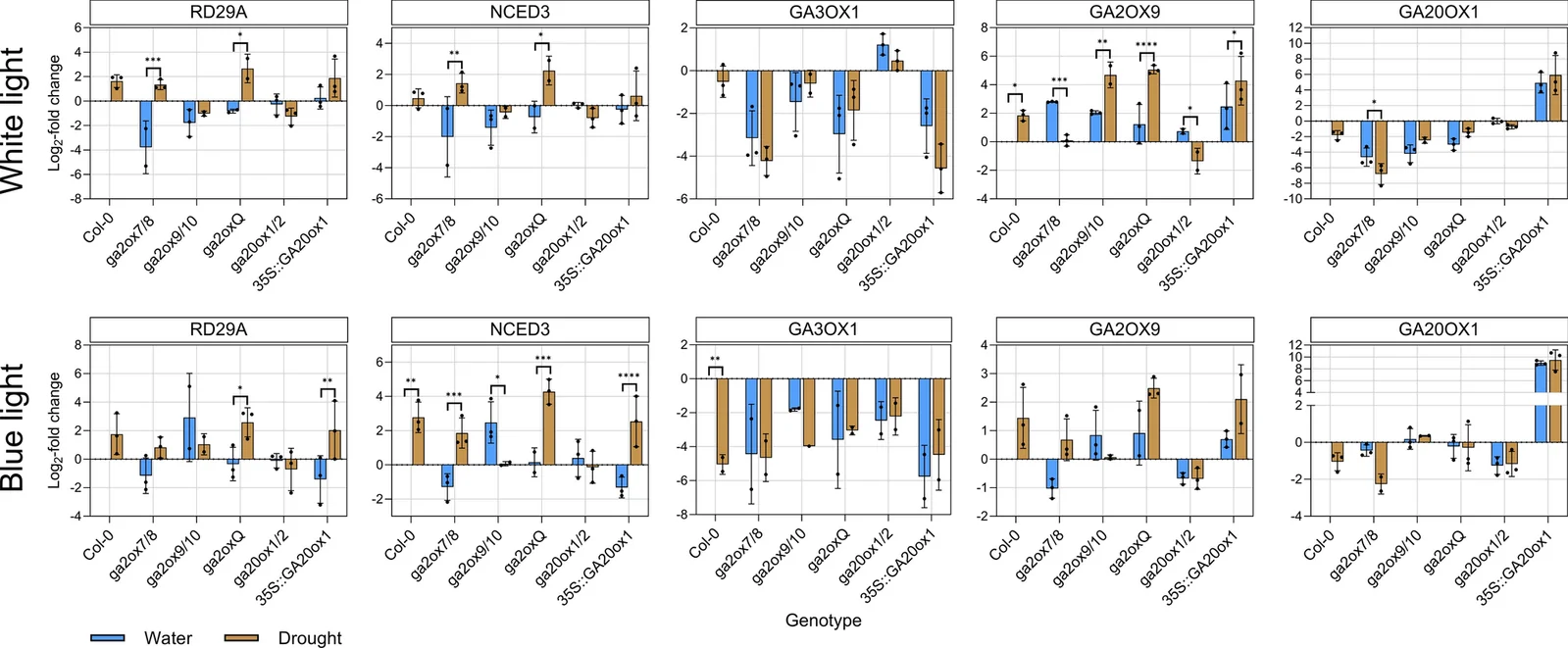
Relative expression of selected genes in leaves of Col-0 and lines with modified GA metabolism, as described in Fig. 6. Transcript levels were determined by RT-qPCR and are plotted relative to watered Col-0 separately for white and blue light. Differences between watering treatments were evaluated separately for each genotype and light condition using planned contrasts derived from the three-way ANOVA (Supplementary file 9 Table S5). *P*-values were adjusted for multiple comparisons using the Holm method. Only significant watering treatments comparisons are indicated by asterisks (*Padj < 0.05, **Padj < 0.01, ***Padj < 0.001, ****Padj < 0.0001)

The response of these lines to water restriction was further investigated by comparing gene expression in shoots of watered and drought-stressed plants under white and blue light. Expression levels of the stress gene *RD29a*, the ABA biosynthesis gene *NCED3,* the GA-biosynthesis genes *GA20ox1* and *GA3ox1*, shown to respond to drought and blue light, respectively, and the drought-responsive GA catabolism gene *GA2ox9* were determined by RT-qPCR. In Fig. 9, expression is shown relative to watered Col-0 in white and blue light, respectively.

In white light, *RD29a* and *NCED3* were similarly up-regulated by drought in Col-0, *ga2ox7/8, ga2oxQ* and *35S::GA20ox1* with slight down-regulation in *ga2ox9/10* and *ga20ox1/2*. In watered plants, the expression of these two genes was lower than in Col-0 in all three *ga2ox* mutants indicating that these lines may be under less stress than watered WT, but there was little change in expression in the GA-deficient *ga20ox1/2* mutant or the GA-over-accumulating *35S::GA20ox1* line suggesting that the stress is not sensitive to overall GA status. The response to drought in blue light was similar to that in white light with up-regulation of *RD29a* in all lines except *ga20ox1/2*, and including *ga2ox9/10.* Expression of *NCED3* behaved as in white light. However, with the exception of *ga2ox7/8,* the strong down-regulation of *RD29a* and *NCED3* expression in watered *ga*2ox lines relative to WT was not apparent.

In white light, the expression of the biosynthetic genes *GA20ox1* and *GA3ox1* was reduced relative to Col-0 in the *ga2ox* mutants under watered and drought-stressed conditions indicating that their regulation is dominated by the homeostasis mechanism. Furthermore, *GA3ox1* expression is modestly up-regulated in *ga20ox1/2* relative to Col-0 and strongly down-regulated in *35S::GA20ox1*. By contrast, *GA2ox9* is up-regulated in the *ga2ox* mutants and *35S::GA20ox1* and more strongly under drought. However, *GA2ox9* was not up-regulated in *ga2ox7/8* when grown under drought. In blue light, expression changes were similar but, in most cases, smaller than in white light. A notable difference is the expression of *GA3ox1* with reduced down-regulation in *ga2ox7/8* and up-regulation rather than down-regulation in *ga2ox9/10* and *ga2oxQ*.

## Discussion

The involvement of GA signalling in stress responses is primarily through the resulting growth adjustment although this effect is less commonly reported for drought responses (Colebrook et al. 2014). Differential growth between roots and aerial organs in response to drought has been associated with changes in GA content (Ptosková et al. 2022). In the current experiments, water restriction did not produce a statistically significant change in bioactive GA_4_ content measured in whole rosette leaves and roots, so changes in this hormone in response to drought may have been restricted to specific tissues, which for technical reasons could not be determined. However, the concentration of GA_34_, a catabolite of GA_4_, decreased in leaves and increased in roots of plants grown under white light during drought stress, indicating a reduced flux through the biosynthetic pathway in shoots and an enhanced flux in roots. Stress responses have been associated with increased GA turnover by 2-oxidation (Colebrook et al. 2014) and indeed transcript expression of *AtGA2ox6* and *AtGA2ox9* is up-regulated in shoots in the drought-stressed plants but much less strongly in roots. However, as was found for wheat (Ptosková et al. 2022), reduced biosynthesis as well as increased catabolism contribute to drought-induced attenuation of GA content in shoots.

Drought stress resulted in a strong increase in the stress-responsive hormone ABA. Although ABA levels in roots were lower than in leaves, they increased more in response to drought and furthermore the concentration of dihydrophaseic acid (DPA), an ABA catabolic product, was more strongly elevated by drought in roots than in leaves, indicating active ABA turnover. The response of roots to ABA concentration and drought is biphasic: a moderate increase in ABA associated with mild drought promotes root growth, whereas high ABA accumulation under more severe drought inhibits root elongation (Li et al. 2017). Therefore, ABA concentrations must be tightly controlled to maintain optimal root growth in a water limiting environment. The higher accumulation of DPA in roots in response to drought may reflect enhanced ABA catabolism to prevent excessive ABA accumulation. Additionally, the ABA turnover could be associated with long-distance transport, contributing to whole plant drought response coordination (Huntenburg 2022).

Drought induced increase in the concentrations of the stress hormones JA and JA-Ile in leaves and SA in both leaves and roots (Fig. S1b, Table S3). Jasmonates have been shown to accumulate in response to drought (de Ollas et al. 2018) and to function in association with ABA (Munoz-Espinoza et al. 2015; de Ollos and Dodd 2016; Wang et al. 2020a). de Ollas et al. (2013) showed that under drought stress, early accumulation of JA was necessary for the subsequent ABA increase in roots. However, JA and SA accumulation may be transitory during prolonged drought, due to ABA-induced suppression (Munoz-Espinoza et al. 2015), which may explain the smaller effect on jasmonate levels in roots, where ABA levels increased more strongly. SA accumulation is associated with improved drought tolerance and inhibition of light-induced stomatal opening in arabidopsis (Okuma et al. 2014). Modification of cytokinin profiles is a well-documented response to drought that may serve to adjust growth distribution and enhance stress tolerance (Nishiyama et al. 2011; Prerostová et al. 2018). *cis*-Zeatin is considered to have a role in stress responses (Schäfer et al. 2015) and we observed drought-induced accumulation of *c*Z, *c*ZR, and their corresponding glucosides in leaves but not in roots. By contrast, there was little effect of drought on the growth-active *t*Z and *t*ZR in leaves with a decrease in roots under white light that is consistent with maintaining root growth (Werner et al. 2010).

Changes in expression of GA metabolism genes in response to drought were more pronounced in leaves than in roots. The expression of *GA20ox1*, the major *GA20ox* gene in Arabidopsis, was down-regulated by water restriction in both leaves and roots and more strongly in white light. Down-regulation of *GA20ox* gene expression in response to abiotic stress, including drought, has been reported for several species and transcription factors mediating this response have been identified (Chen et al. 2019; Song et al. 2021). Constitutive expression of *AtGA20ox1* in Arabidopsis was shown by Kudo et al. (2019) to partially restore growth when co-expressed with *DREB1a* without compromising the drought tolerance imparted by this transcription factor. Under our conditions, the *AtGA20ox1*-overexpressing plants were significantly smaller than controls under drought in white light conditions, indicating that the greater stress experienced by these plants out-weighed potential growth stimulation. Plants lacking both *ga20ox1* and *ga20ox2* were not further stunted under drought and, on the basis of *RD29a* and *NCED3* expression as well as changes in leaf temperature and relative water content, these plants did not exhibit signs of advanced stress, consistent with reduced water usage.

Expression of *GGPPS* genes was reduced by drought under white light, with two genes down-regulated in leaves and five genes strongly down-regulated in roots. This could contribute to the reduced GA content, although it is unclear which GGPPS paralogue provides substrate for GA biosynthesis (Hedden 2020). *GGPPS* transcript levels were reduced less by drought under blue light, particularly in roots, suggesting an enhanced flux into the GGPP precursor pool. These results indicate an increased capacity for GGPP metabolism in drought-stressed roots under blue light, which is not limited to GA biosynthesis. In addition, up-regulation of *GA20ox2* and *GA3ox2* expression under blue light, particularly in stressed plants, may buffer GA content against drought-induced reduction, as found in our analysis (Fig. 2).

Enhanced GA catabolism makes a major contribution to the reduced GA content in response to stress (Colebrook et al. 2014). Two *GA2ox* catabolism genes, *GA2ox6* and *GA2ox9* were up-regulated in shoots under drought in white light. In contrast to wheat seedlings, where differential *GA2ox* expression between leaves and roots was reported (Ptosková et al. 2022), the *GA2ox* expression profile was similar in both organs in Arabidopsis, although the C_20_-GA2ox genes were expressed more highly in roots than in shoots. Drought resulted in much weaker up-regulation of *GA2ox6* and *GA2ox9* in roots than in shoots whilst other catabolism genes, including *GA2ox8* and *CYP714A1* were down-regulated, contributing to the drought-induced promotion of GA production in roots.

Whilst expression of some GA metabolism and signalling genes may respond directly to the drought stress, other genes are regulated indirectly via homeostasis in response to an altered GA content. In contrast to *GA2ox6* and *GA2ox9*, *GA2ox1* and *GA2ox2* were down-regulated by drought. Both genes respond strongly to GA status (Rieu et al. 2008a). Expression of *GA3ox1*, the major *GA3ox* gene in leaves and roots, was slightly up-regulated in shoots in response to drought whilst RNA-seq indicated it was down-regulated in roots although this was not confirmed by RT-qPCR (Fig. 9). Expression of *GA3ox1* is feedback regulated (Cowling et al. 1998) as confirmed in the current work by its increased expression in shoots of the GA-deficient *ga1-3* mutant relative to Col-0 (Fig. 5). However, in contrast to *GA20ox1*, in which expression is up-regulated with respect to Col-0 in both roots and shoots of *ga1-3*, *GA3ox1* expression was not elevated in *ga1-3* roots suggesting that its expression is not under homeostatic regulation in Arabidopsis roots. Differential regulation of *GA3ox1* and *GA20ox1* expression between shoots and roots was also noted for the ABA-deficient *aba2* mutant. *GA20ox1* expression was elevated in *aba2* roots consistent with lack of *GA20ox1* suppression by the ABA- and drought-inducible ATHB12 transcription factor (Son et al. 2010). *GA3ox1* was down-regulated in *aba2* roots relative to Col-0 as was also noted by Son et al. (2010) in inflorescence stems of *athb12*.

The influence of C_19_- and C_20_-GA2ox enzymes on drought tolerance was investigated using mutants and their influence compared with the GA-deficient mutant *ga20ox1/2* (Rieu et al. 2008b) and a GA-over-producing line *35S::AtGA20ox1* (Coles et al. 1999). Despite low expression of genes encoding the C_20_-GA2ox enzymes GA2ox7 and GA2ox8 in shoots, loss of both of these genes had a relatively large effect on growth under normal conditions and on drought tolerance. The leaf temperature increase in drought-stressed plants for the *ga2ox7/8* mutant was less than for Col-0, *35S::GA20ox1* and other *ga2ox* mutants under both light conditions suggesting compromised ability to adjust stomatal aperture. On the basis of a GUS reporter, *AtGA2ox8* was shown to be expressed in stomatal guard cells (Li et al. 2019) as was *SlGA2ox7* in tomato (Shohat et al. 2021). Expression of *SlGA2ox7*, which is the closest tomato orthologue of *AtGA2ox8*, was induced by drought in guard cells in parallel with reduced GA content, whilst stomatal movement in response to drought was reduced in an *slga2ox7* mutant (Shohat et al. 2021). As reported by Shohat et al. (2020), accumulation of the DELLA protein PROCERA in a low GA environment promotes stomatal closure through enhanced ABA transport into guard cells. We could detect no effect of drought on *AtGA2ox8* transcript levels in shoots but these levels were very low and localised changes in guard cells may have been masked by broader changes in the leaf. *AtGA2ox8* was more highly expressed in roots, in which it was strongly down-regulated by drought. As for the *GA20ox1*-overexpressing line, none of the *ga2ox* mutants promoted shoot growth relative to Col-0 under drought suggesting that reduced GA content is not a major cause of the drought-imposed growth restriction under the experimental conditions but may contribute to drought tolerance.

Light conditions have a major impact on plant responses to stress including drought (Roeber et al. 2021). Blue light through the PHOT and CRY receptors influences the response to drought. Low fluence blue light promotes stomatal opening primarily through the PHOT receptors (Kinoshita et al. 2001), although the CRY receptors also play a role through suppression of COP1 (Mao et al. 2005; Kang et al. 2009). Accordingly, Arabidopsis *cry1/cry2* double mutants show reduced BL-induced stomatal opening, which promotes drought tolerance, whilst plants overexpressing *myc-AtCRY1* and *myc-AtCRY2* were more sensitive to drought (Mao et al. 2005). In the present study, plants growing under continuous low fluence blue light were slightly smaller with reduced root mass compared to plants grown in short-day white light. The reduction in rosette leaf areas and mass under drought was smaller under blue light, associated with reduced water loss. It is notable that under well-watered conditions the leaf temperatures of the GA-deficient *ga20ox1/2* was lower in blue light than in white light consistent with higher stomatal conductance in blue light. However, with the exception of *ga20ox1/2* and *aba2*, leaf temperatures of well-watered plants were similar under white and blue light. Under drought, leaf temperature increased less under blue light, particularly in the *ga2ox* mutants. This may reflect reduced stomatal responsiveness rather than lower drought severity, as RWC decreased more in some mutants (*ga2ox7/8*, *ga2ox9/10* and *ga2ox1,2,3,4,6*) under blue light than under white light, suggesting impaired stomatal regulation under water restriction. Indeed, up-regulation of the stress gene *RD29a* by drought was > 2-fold greater in white light than in blue light in both organs. Furthermore, with some exceptions, changes in expression of GA and ABA metabolism and signalling genes due to drought were greater under white light. However, in the case of GA biosynthesis genes, such as *GA20ox2* in shoots and *GA3ox2* in roots, drought-induced transcript changes in blue light may be compensated by the strong up-regulation by blue light under drought. Overall, these findings suggest that plants were experiencing less stress under blue light, indicating an improved capacity to adapt to water-deficit conditions.

## Summary and conclusions

ABA levels increased in both leaves and roots in response to water restriction, with a stronger accumulation in roots, irrespective of light conditions. These relative changes in ABA levels were associated with differential expression of ABA-biosynthetic *NCED* genes. In contrast to ABA, GA levels changed more modestly with no significant difference in GA_4_ in response to drought, whilst the concentration of the GA_34,_ catabolite GA_4_, decreased in leaves but increased in roots under white light, consistent with changes in biosynthesis rather than catabolism contributing to the altered GA content. In drought-stressed shoots under white light, the expression of the rate-limiting GA-biosynthesis *GA20ox* genes was reduced. By contrast, the C_19_-GA2ox gene *AtGA2ox6* and the C_20_-GA2ox gene *AtGA2ox9* accumulated higher transcript levels, whereas the other *GA2ox* genes were generally down-regulated. In roots, there was slight up-regulation of *AtGA2ox6* and *AtGA2ox9* under drought, with strong down-regulation of several other *GA2ox* genes including *AtGA2ox8*. Continuous blue light mitigated the drought-induced changes in GA content observed under white light by buffering *GA20ox* expression through up-regulation of *AtGA20ox2* in drought-stressed shoots and both stressed and watered roots. In general, based on molecular and physiological data, plants appeared less stressed under the blue light condition than in white light.

Transcript analysis in ABA- and GA-deficient mutants indicated ABA-dependent regulation of *AtGA20ox1* in roots, and feedback regulation of *AtGA20ox1* in both leaves and roots, whereas *AtGA3ox1* responded to GA-deficiency by up-regulation only in leaves. Leaf temperature measurements indicated an enhanced response to drought of GA2ox mutants and the *GA20ox1* over-expression line, except for the *ga2ox7/8* double mutant, in which the capacity to regulate stomatal movement may be impaired. This is consistent with the reported expression of *AtGA2ox8* and its tomato orthologue in stomatal guard cells.

## Supplementary Information

Below is the link to the electronic supplementary material.

Supplementary file1 (DOCX 15 KB)

Supplementary file2 (XLSX 20902 KB)

Supplementary file3 (XLSX 38 KB)

Supplementary file4 (PDF 1444 KB)

Supplementary file5 (DOCX 15 KB)

Supplementary file6 (XLSX 14 KB)

Supplementary file7 (XLSX 29 KB)

Supplementary file8 (DOCX 22 KB)

Supplementary file9 (XLSX 24 KB)

## Data Availability

The datasets generated and analysed during the current study are publicly available in the Zenodo repository with a https://doi.org/10.5281/zenodo.21672515. The raw RNA sequencing data have been deposited in the NCBI Sequence Read Archive (SRA) under BioProject accession number PRJNA1504113.

## Abbreviations

CRY: CRYPTOCHROME
*c*Z: *cis*-Zeatin
ga2oxQ: ga2ox1,2,3,4,6 quintuple mutant
GA: Gibberellin
GID1: GIBBERELLIN-INSENSITIVE DWARF 1
JA: Jasmonic acid
PHOT: PHOTOTROPIN
NCED: 9-*cis*-Epoxycarotenoid dioxygenase
SA: Salicylic acid
SD: Short days
*t*Z: *trans*-Zeatin
