# Supplementary material for: Light modulates organ-specific gibberellin–abscisic acid crosstalk during drought response in Arabidopsis thaliana
Source: Planta. 2026 Oct 17;264(6):172. doi: 10.1007/s00425-026-05204-7 (PMC13676654; doi:10.1007/s00425-026-05204-7)
Supplement: Supplementary file 1 [file 425_2026_5204_MOESM4_ESM.pdf]

Light modulates organ-specific gibberellin-abscisic acid crosstalk during drought response in *Arabidopsis thaliana*

Authors: Tereza Miksteinová[1,2], Marek Szecówka[2,3], Ivan Petřík[1,2], Terezie Urbanová[1], Tereza Trávníčková[2], Martina Špundová [4], Martin Fellner[1,2], Peter Hedden[1,2,5]

[1] Laboratory of Growth Regulators, Institute of Experimental Botany, Czech Academy of Sciences, Šlechtitelů 27, CZ-77900 Olomouc, Czech Republic; [2] Laboratory of Growth Regulators, Faculty of Science, Palacký University, Šlechtitelů 27, 77900 Olomouc, Czech Republic; [3] Centre of Plant Structural and Functional Genomics, Institute of Experimental Botany, Czech Academy of Sciences, Olomouc, Czech Republic; [4] Department of Biophysics, Faculty of Science, Palacký University, Olomouc, Czech Republic; [5] Rothamsted Research, Harpenden AL5 2JQ, UK

Corresponding author: Peter Hedden hedden@ueb.cas.cz, ORCID: 0000-0002-4216-7488

Supplementary Figures

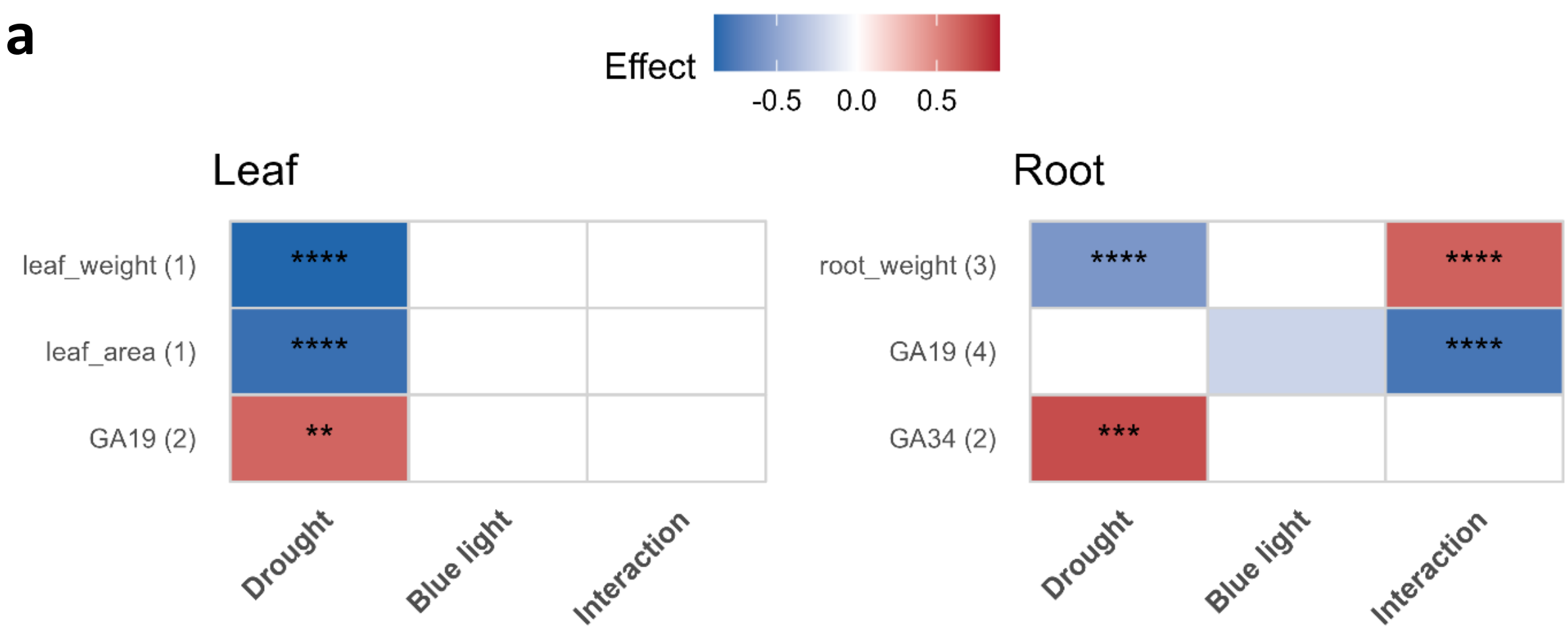

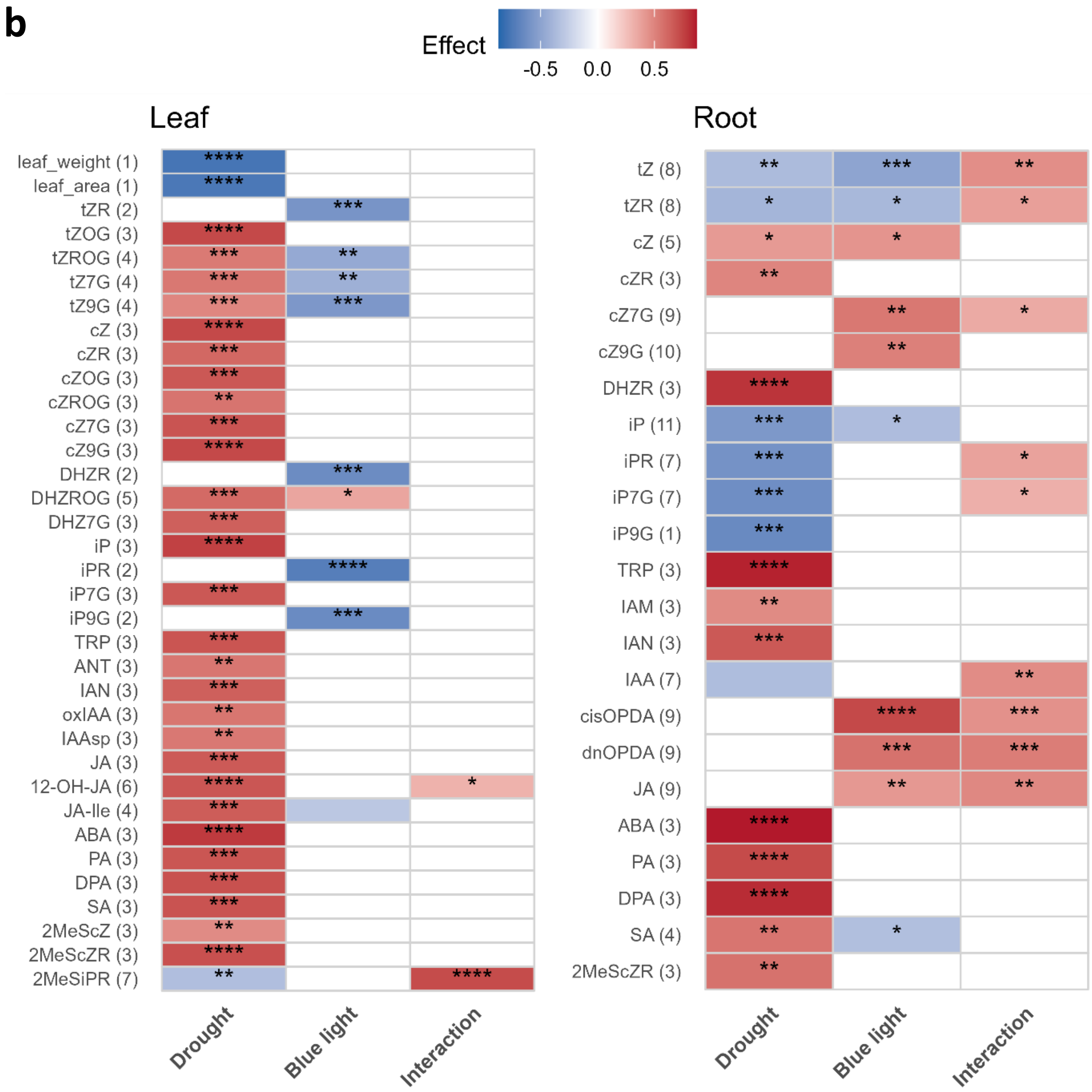

Fig. S1 Multivariate analysis of growth parameters and hormone concentrations. The heatmap shows the effects of drought, blue light, and their interaction on root weight, leaf weight and size and concentrations of GAs (**a**) and other hormones (**b**). Colours represent effect sizes estimated as coefficients of a multiple linear model. Blue indicates a negative effect, red indicates a positive effect, and white indicates no detectable effect. Asterisks denote statistical significance (blank $P \geq 0.1$, * $P < 0.05$, ** $P < 0.01$, *** $P < 0.001$, **** $P < 0.0001$). Drought repression (1), Blue-light repression (2), Drought induction (3), Independent drought induction and blue-light repression (4), Independent drought and blue-light induction (5), Blue-light-enhanced drought induction (6), Blue-light-attenuated drought repression (7), Mutual-attenuated repression (8), Drought-enhanced blue-light induction (9), Blue-light induction (10), Independent drought and blue-light repression (11). GA_4, gibberellin A4; GA_{34}, gibberellin A_{34}; GA19, gibberellin A_{19}; *t*Z, trans-zeatin; *t*ZR, trans-zeatin riboside; *t*ZOG, trans-zeatin *O*-glucoside; *t*ZROG, *trans*-zeatin riboside *O*-glucoside; *t*Z7G, trans-zeatin 7-glucoside; *t*Z9G, *trans*-zeatin 9-glucoside; *c*Z, *cis*-zeatin; *c*ZR, *cis*-zeatin riboside; *c*ZOG, *cis*-zeatin *O*-glucoside; *c*ZROG, *cis*-zeatin riboside *O*-glucoside; *c*Z7G, *cis*-zeatin 7-glucoside; *c*Z9G, *cis*-zeatin 9-glucoside; DHZ, dihydrozeatin; DHZR, dihydrozeatin riboside; DHZOG, dihydrozeatin O-glucoside; DHZROG, dihydrozeatin riboside O-glucoside; iP, isopentenyladenine; iPR, isopentenyladenine riboside; iP7G,

isopentenyladenine 9-glucoside; 2MeScZR, 2-methylthio-cis-zeatin riboside; 2MeScZ, 2-methylthio-cis-zeatin; mTRP, methyltryptophan; TRP, tryptophan; ANT, anthranilic acid; IAM, indole-3-acetamide; IAA, indole-3-acetic acid; IAA-Ala, IAA-alanine; IAA-Asp, IAA-aspartate; IAA-Glu, IAA-glutamate; oxIAA, oxidized indole-3-acetic acid; IBA, indole-3-butyric acid; 9,10-DHJA, 9,10-dihydrojasmonic acid; 12-OH-JA, 12-hydroxyjasmonic acid; JA-Ile, jasmonoyl-isoleucine; ABA, abscisic acid; PA, phaseic acid; DPA, dihydrophaseic acid; SA, salicylic acid.

a

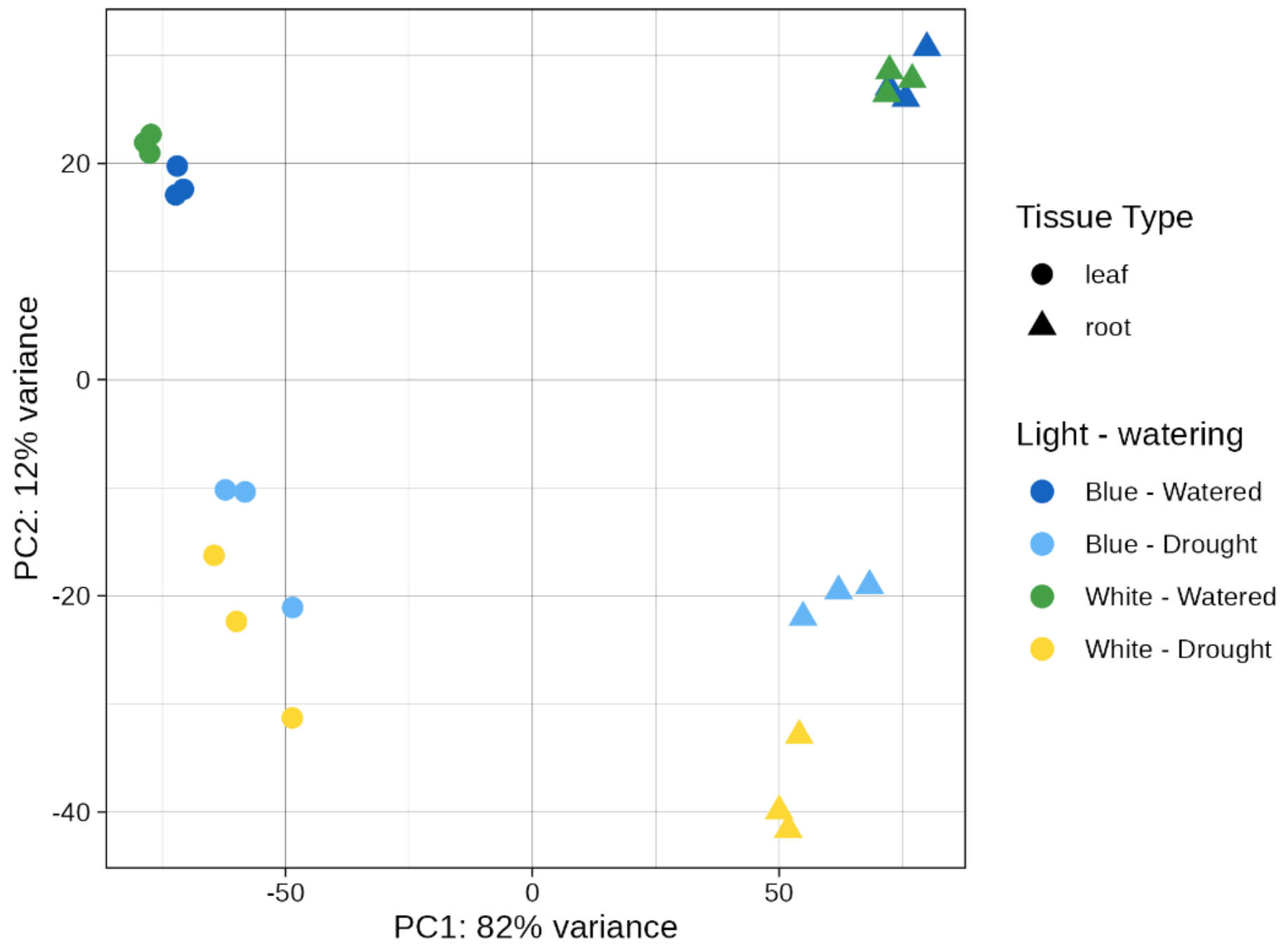

b

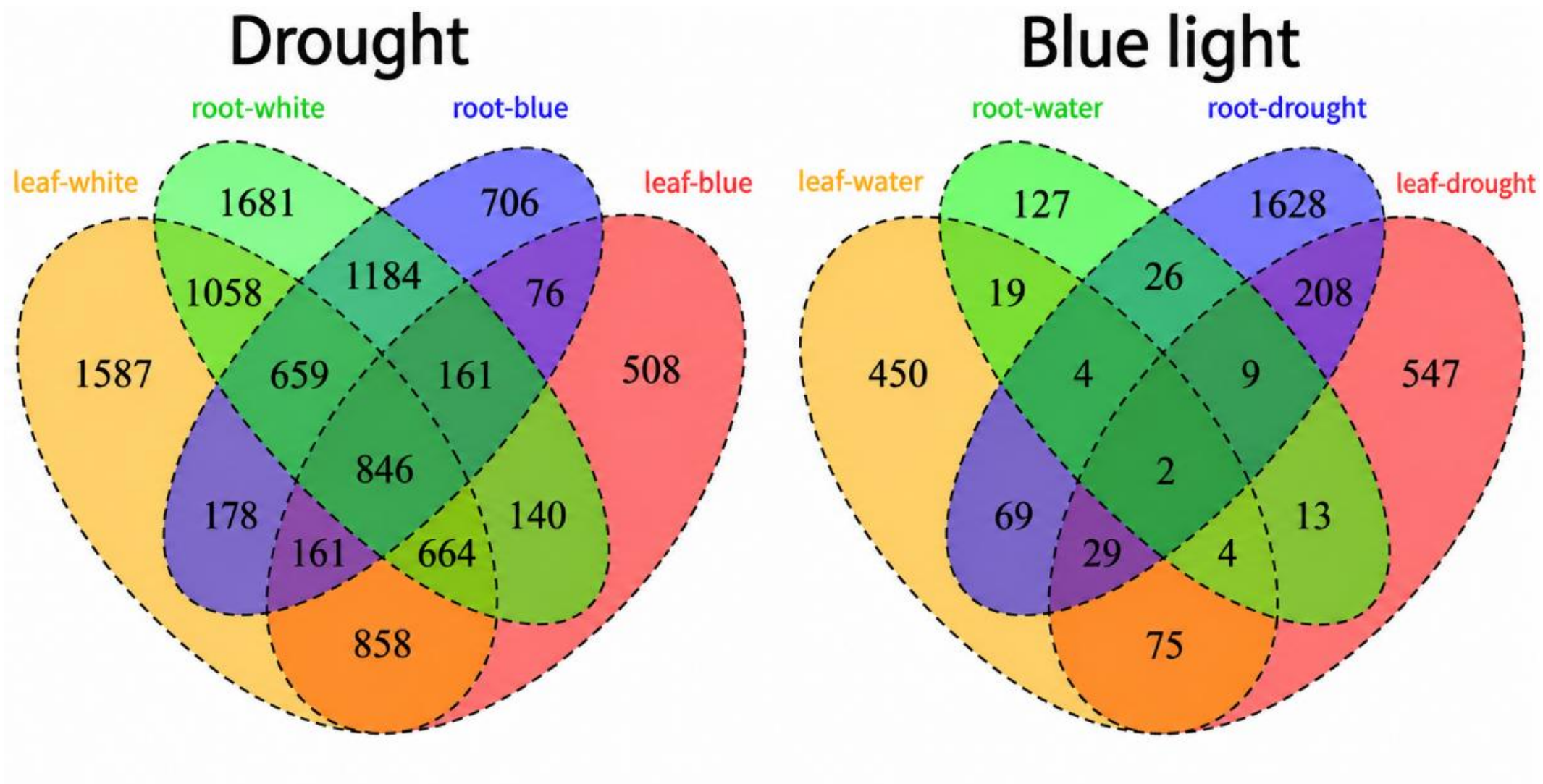

Fig. S2 Principal component analysis of differentially expressed genes (**a**) and Venn diagrams (**b**) showing differentially regulated genes for shoots and roots between watered and droughted and white and blue light.

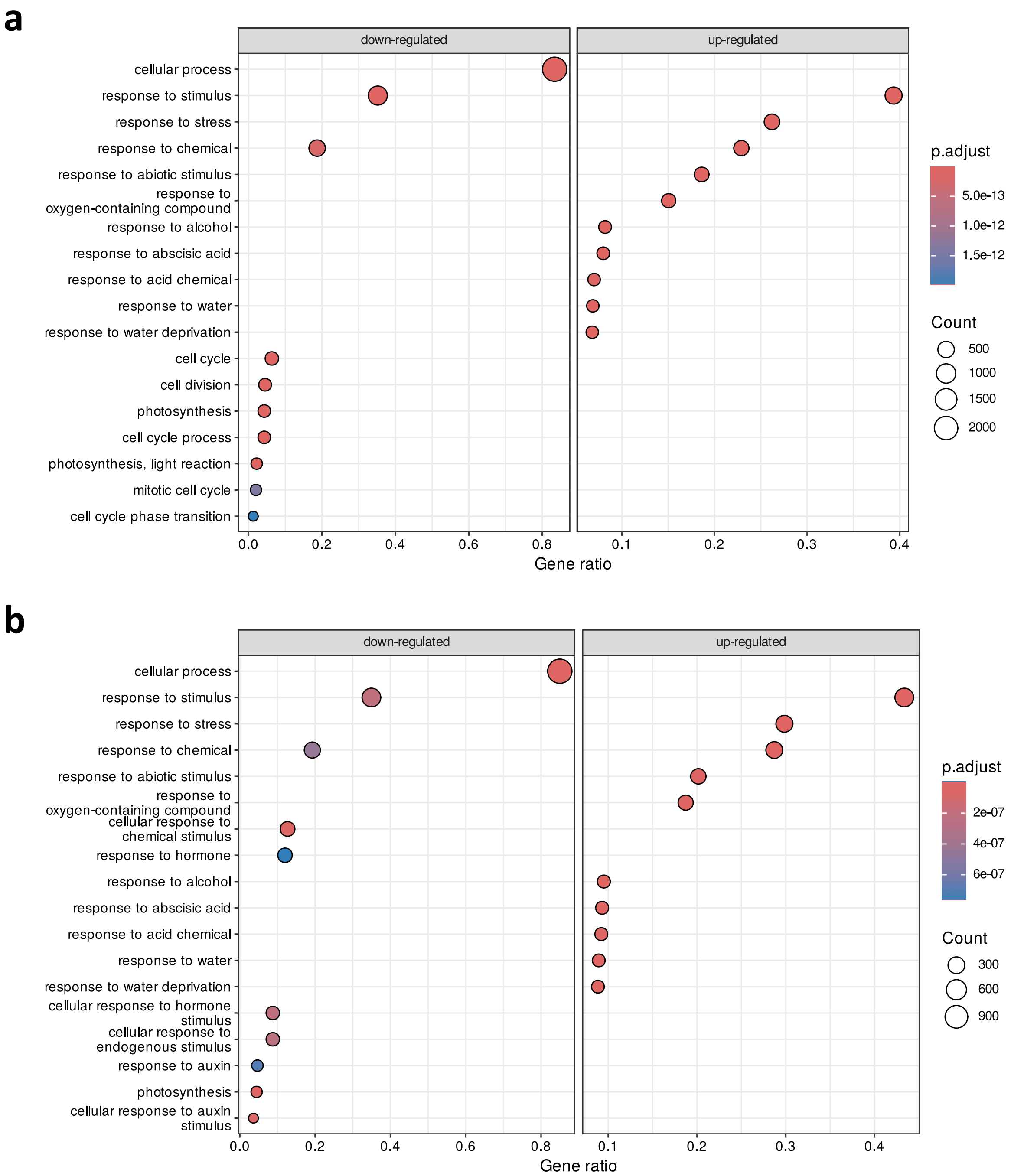
a
down-regulated
up-regulated
cellular process
response to stimulus
response to stress
response to chemical
response to abiotic stimulus
response to oxygen-containing compound
response to alcohol
response to abscisic acid
response to acid chemical
response to water
response to water deprivation
cell cycle
cell division
photosynthesis
cell cycle process
photosynthesis, light reaction
mitotic cell cycle
cell cycle phase transition
0.0
0.2
0.4
0.6
0.8
0.1
0.2
0.3
0.4
Gene ratio
p.adjust
5.0e-13
1.0e-12
1.5e-12
Count
500
1000
1500
2000
b
down-regulated
up-regulated
cellular process
response to stimulus
response to stress
response to chemical
response to abiotic stimulus
response to oxygen-containing compound
cellular response to chemical stimulus
response to hormone
response to alcohol
response to abscisic acid
response to acid chemical
response to water
response to water deprivation
cellular response to hormone stimulus
cellular response to endogenous stimulus
response to auxin
photosynthesis
cellular response to auxin stimulus
0.0
0.2
0.4
0.6
0.8
0.1
0.2
0.3
0.4
Gene ratio
p.adjust
2e-07
4e-07
6e-07
Count
300
600
900

c

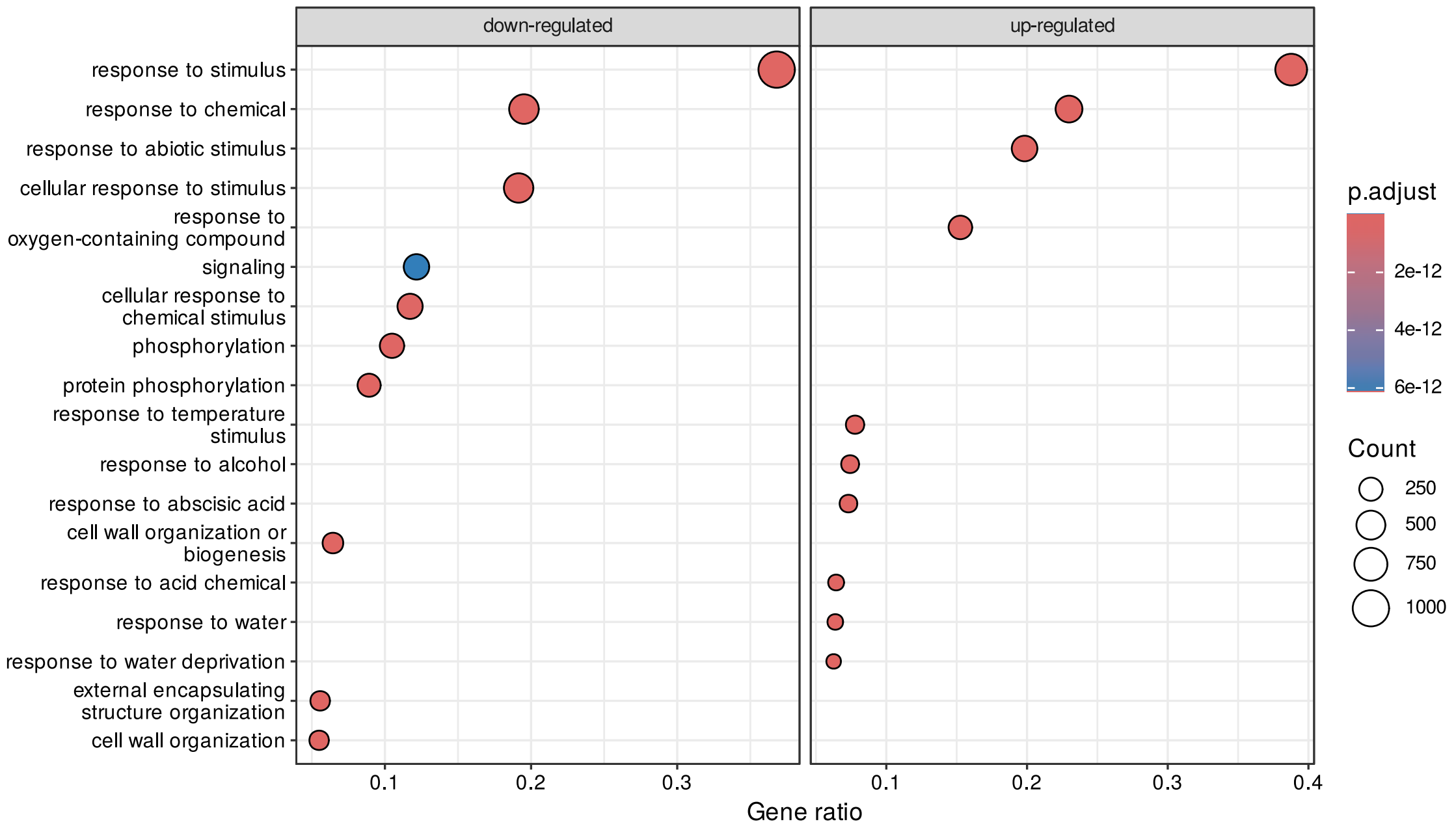

d

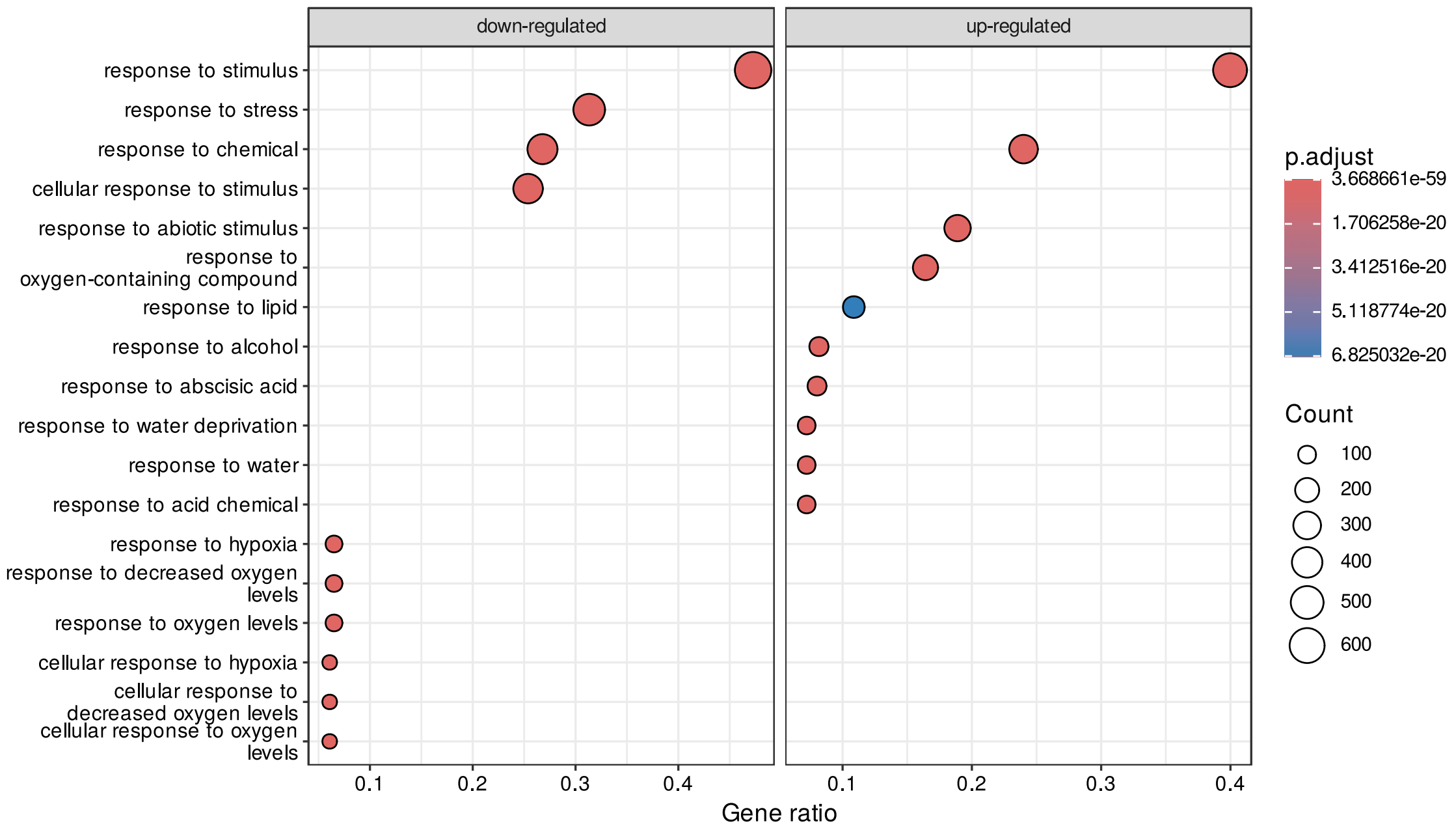

Fig. S3a Gene Ontology (GO) over-representation analysis (ORA) of differentially expressed genes across tissues and conditions. Dot plots show significantly enriched GO Biological Process (BP) terms identified by ORA separately for (**a**) leaf white, (**b**) leaf blue, (**c**) root white, and (**d**) root blue conditions. For each condition, enrichment was performed independently on up-regulated and down-regulated genes passing the defined differential expression thresholds. Each dot represents a GO term; dot size indicates the number of genes associated with the term (Count), and color corresponds to the adjusted p-value (FDR). The x-axis shows the gene ratio, defined as the proportion of differentially expressed genes annotated to a given GO term. Only the top 10 enriched GO terms per regulation category are shown in each panel.

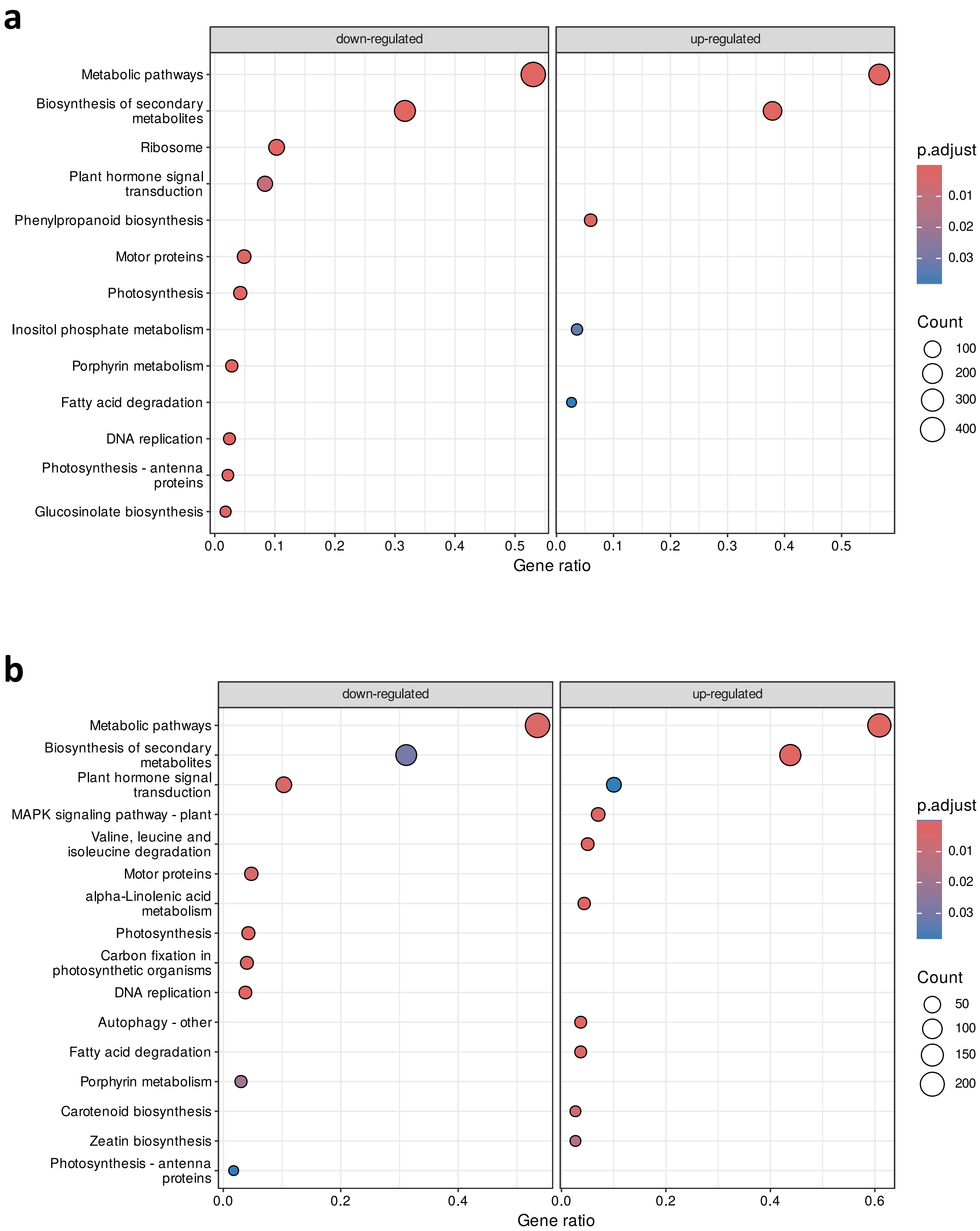
a
down-regulated
up-regulated
Metabolic pathways
Biosynthesis of secondary metabolites
Ribosome
Plant hormone signal transduction
Phenylpropanoid biosynthesis
Motor proteins
Photosynthesis
Inositol phosphate metabolism
Porphyrin metabolism
Fatty acid degradation
DNA replication
Photosynthesis - antenna proteins
Glucosinolate biosynthesis
0.0 0.1 0.2 0.3 0.4 0.5
Gene ratio
p.adjust
0.01
0.02
0.03
Count
100
200
300
400
b
down-regulated
up-regulated
Metabolic pathways
Biosynthesis of secondary metabolites
Plant hormone signal transduction
MAPK signaling pathway - plant
Valine, leucine and isoleucine degradation
Motor proteins
alpha-Linolenic acid metabolism
Photosynthesis
Carbon fixation in photosynthetic organisms
DNA replication
Autophagy - other
Fatty acid degradation
Porphyrin metabolism
Carotenoid biosynthesis
Zeatin biosynthesis
Photosynthesis - antenna proteins
0.0 0.2 0.4 0.6
Gene ratio
p.adjust
0.01
0.02
0.03
Count
50
100
150
200

c

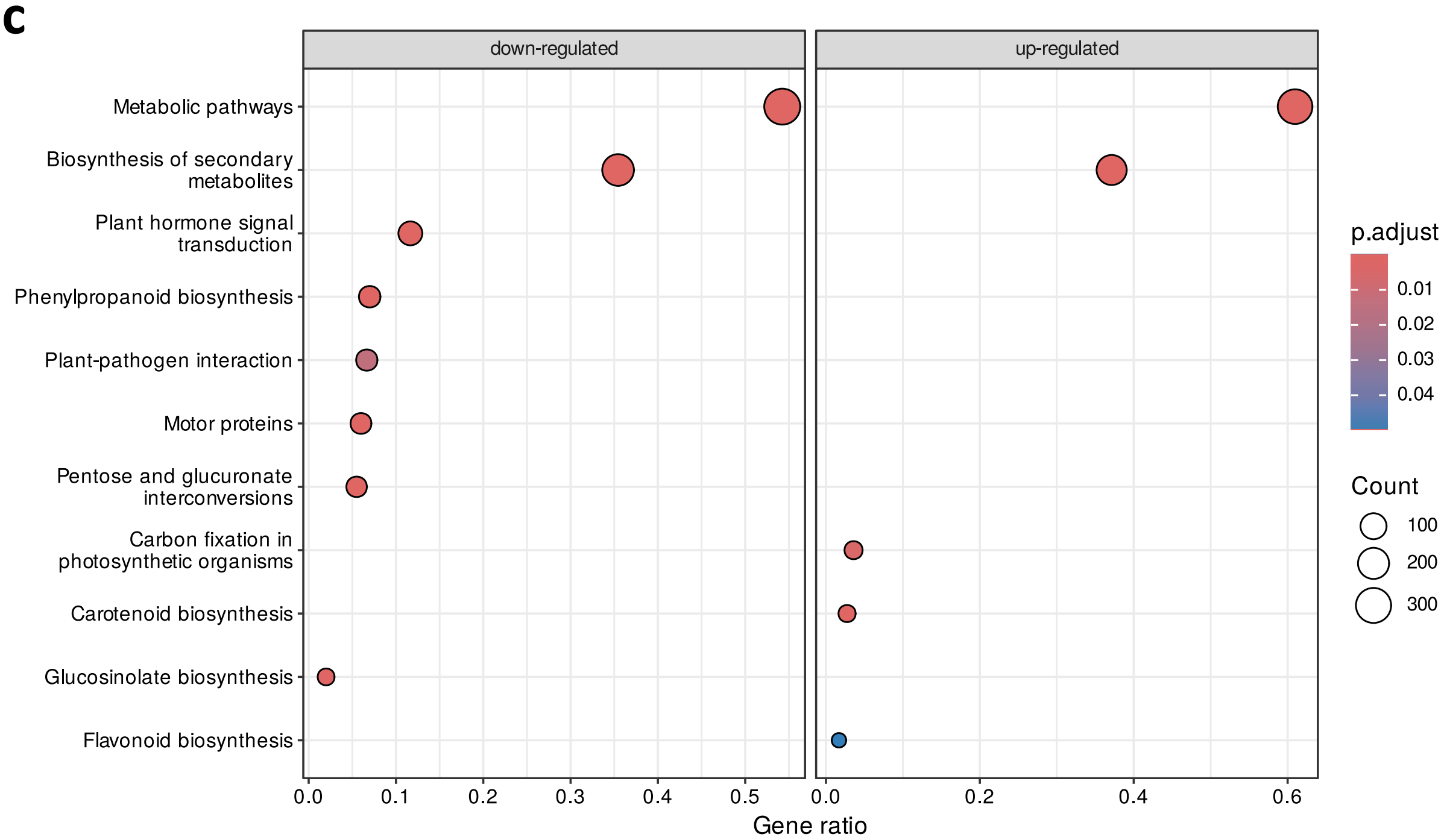

d

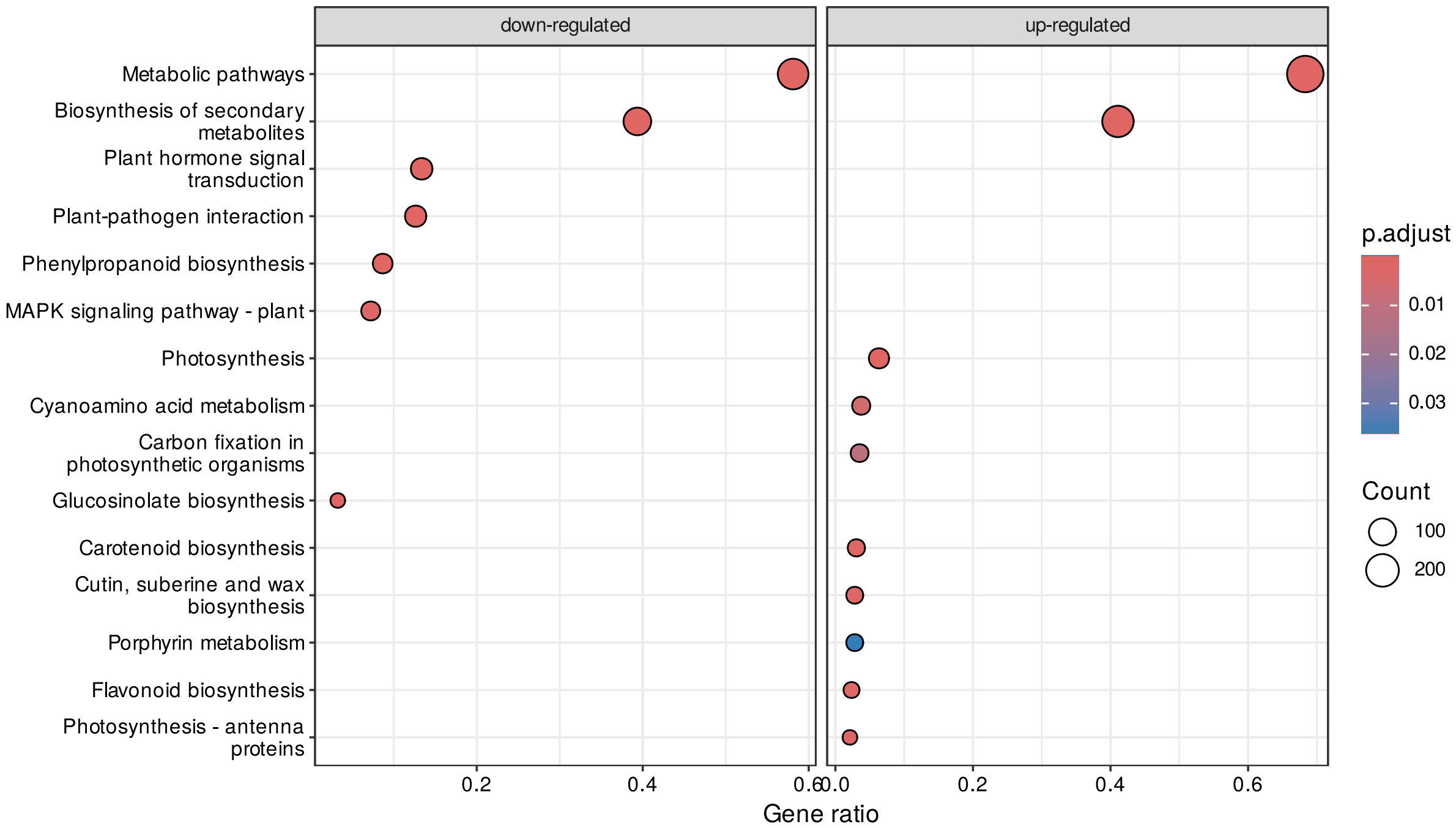

Fig. S3b Gene Ontology (GO) over-representation analysis (ORA) of differentially expressed genes across tissues and conditions. Dot plots show significantly enriched GO KEGG pathway over-representation analysis of differentially expressed genes in leaf and root tissues under white and blue light.ORA dot plots are shown for (**a**) leaf white, (**b**) leaf blue, (**c**) root white, and (**d**) root blue.

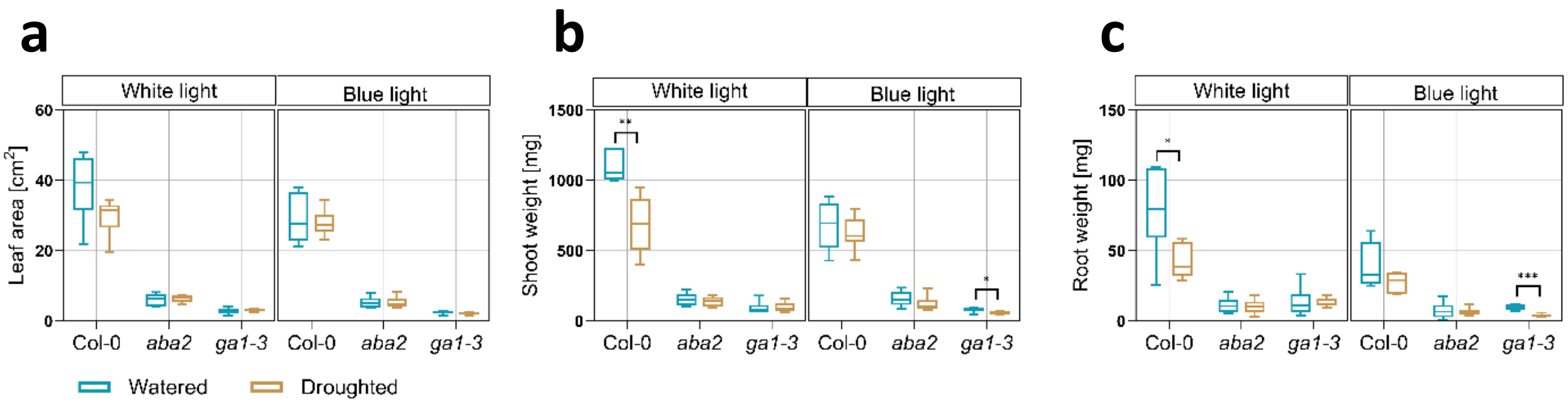

Fig. S4 Growth of 5-week-old *Arabidopsis thaliana* plants (Col-0, *aba2* and *ga1-3)* affected by water restriction, grown in SD white light and continuous blue lights. Rosette areas 7 days after watering was discontinued (**a**). Shoot (**b**) and root weights after 7 days (**c**). Differences between watering treatments were evaluated by using two-sided Welch's t-tests. Only significant watering treatments comparisons are indicated by asterisk (* $P < 0.05$, * $P < 0.01$, ***$P < 0.001$).

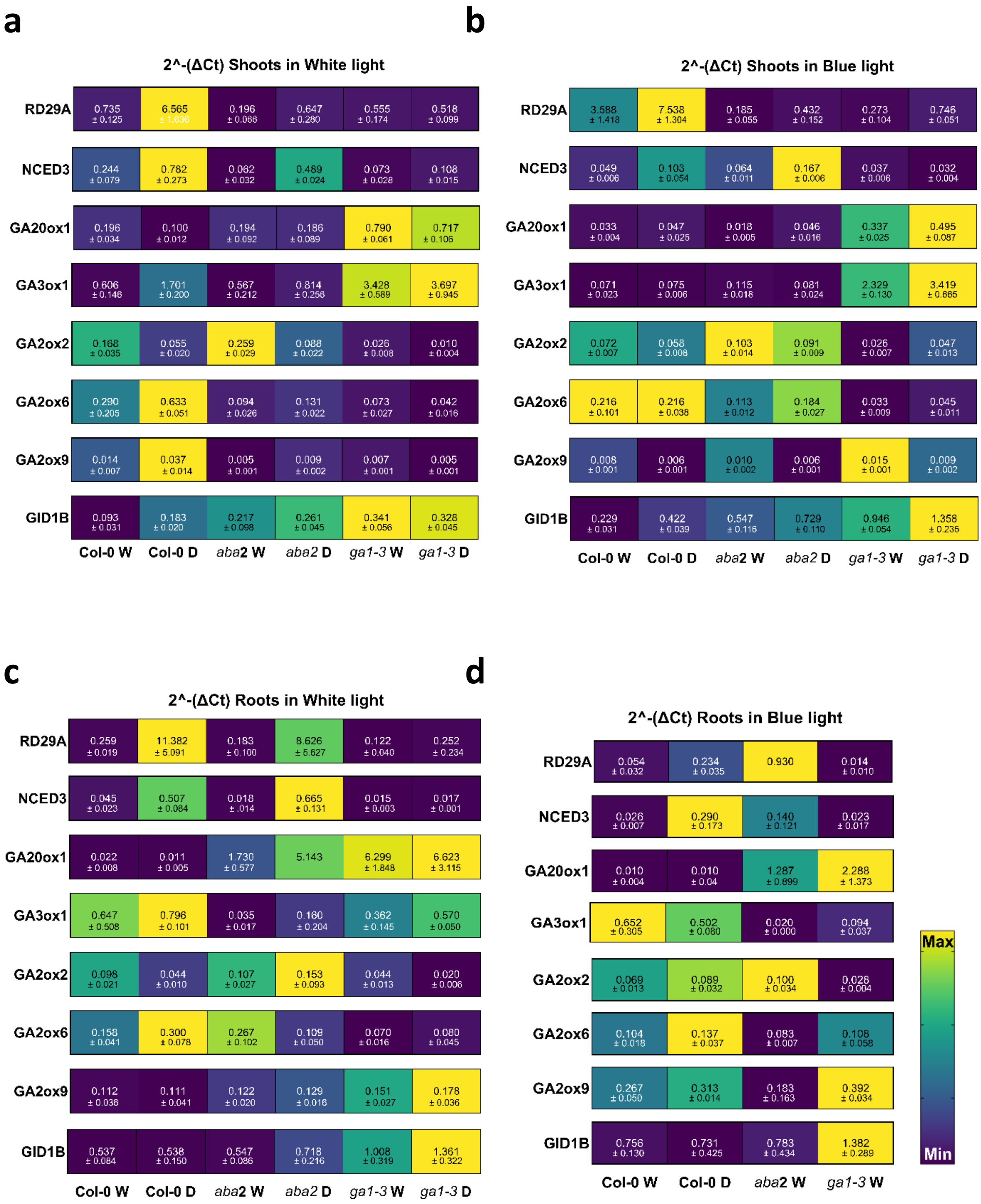

Fig. S5 Relative expression of selected genes (2^-(ΔCt) values) in shoots and roots of Col-0, *aba2* and *ga1-3* comparing plants grown in watered white light (**a**), droughted white light (**b**), watered blue light (**c**) and droughted blue light (**d**).

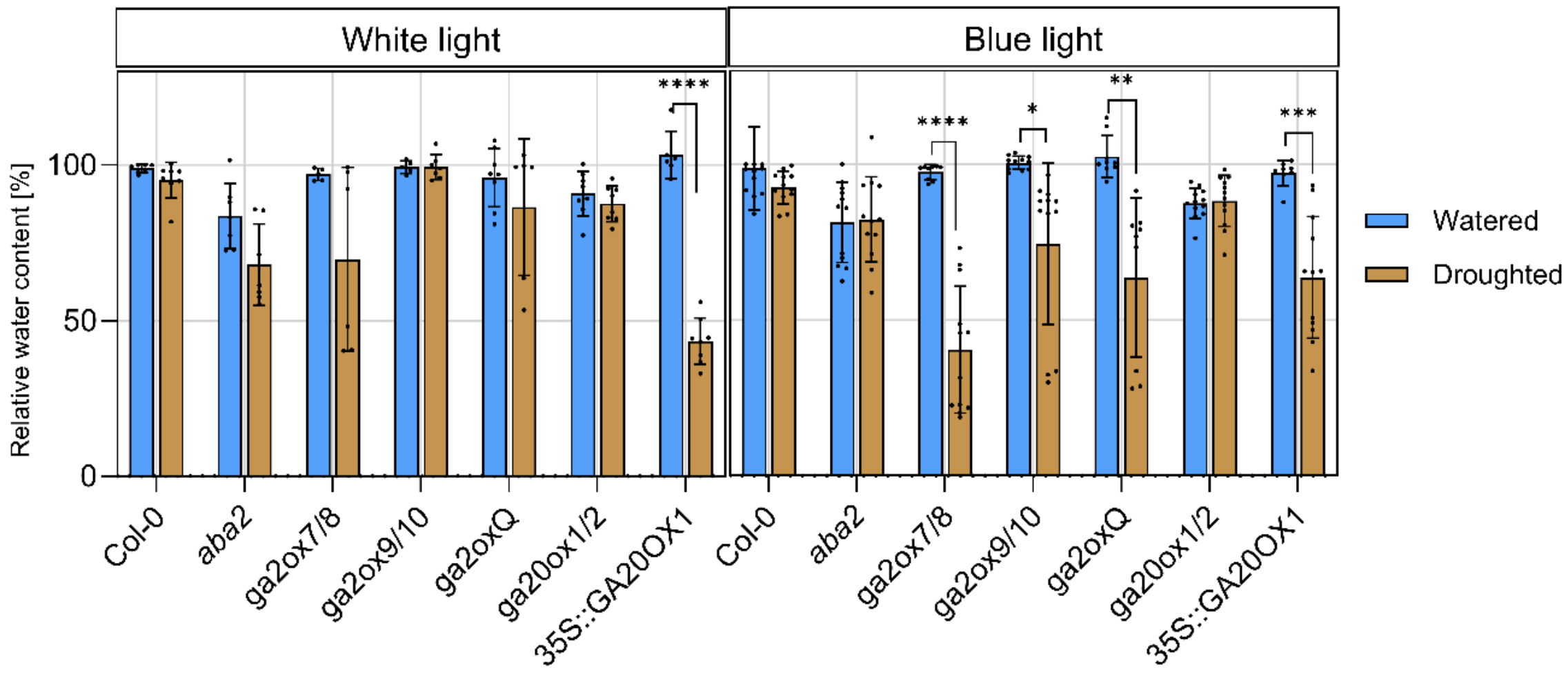

Fig. S6 Relative water content of Col-0, *aba2* and lines with altered GA metabolism in watered white light (**a**), droughted white light (**b**), watered blue light (**c**) and droughted blue light (**d**). Differences between watering treatments were evaluated by using two-sided Welch's t-tests. Only significant watering treatments comparisons are indicated by asterisk (* *P* < 0.05, * *P* < 0.01, ****P* < 0.001, **** *P* < 0.0001).

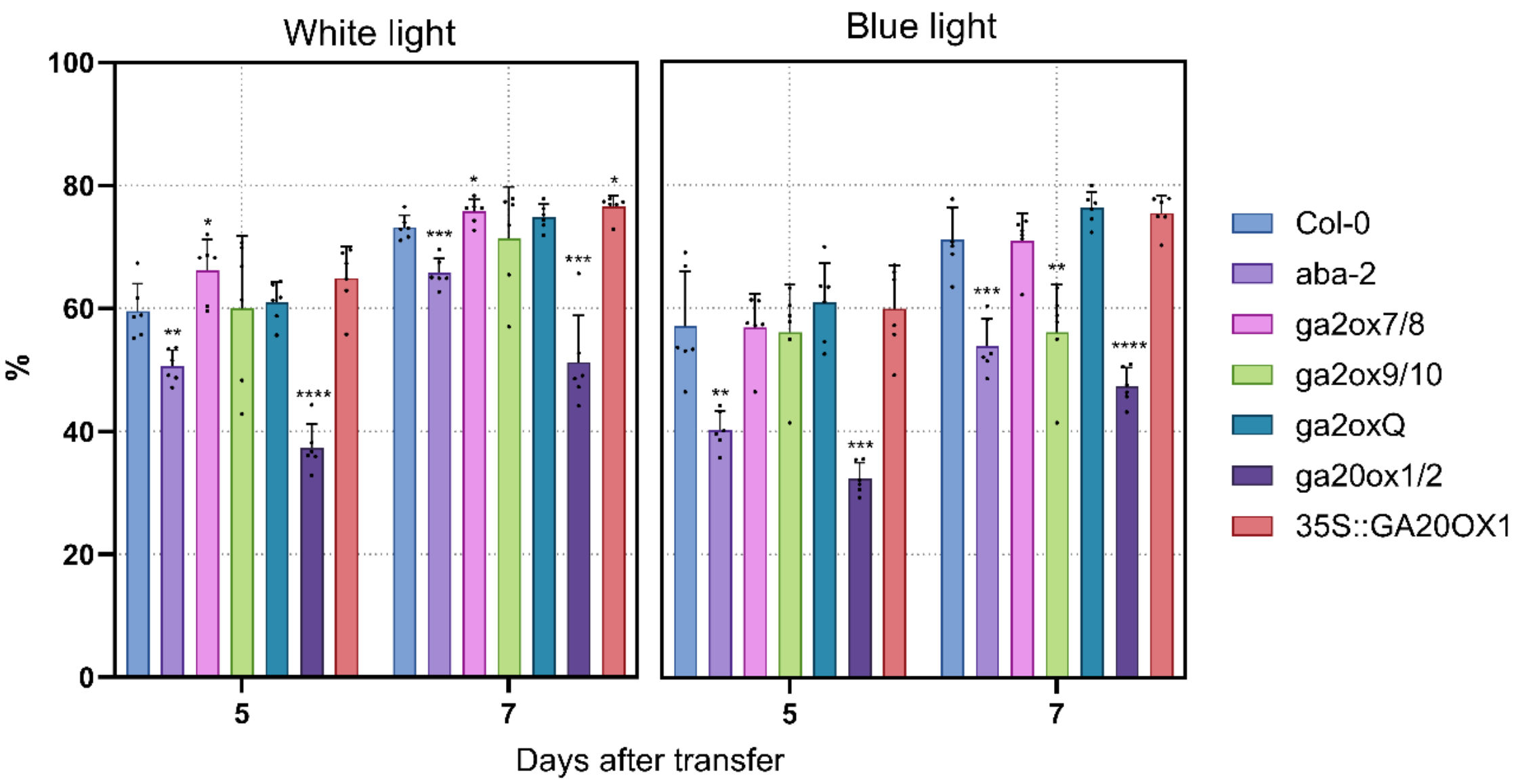

Fig. S7 Water loss from pots with Col-0, *aba2* and lines with altered GA metabolism in white and blue light 5 and 7 days after cessation of watering. Differences between genotypes were evaluated separately for each light condition and time point. Each genotype was compared with Col-0 using a two-sided Welch's *t*-test. Only statistically significant differences are indicated by asterisk (* $P < 0.05$, * $P < 0.01$, ***$P < 0.001$, **** $P < 0.0001$)
