# Supplementary material for: Light modulates organ-specific gibberellin–abscisic acid crosstalk during drought response in Arabidopsis thaliana
Source: Planta. 2026 Oct 17;264(6):172. doi: 10.1007/s00425-026-05204-7 (PMC13676654; doi:10.1007/s00425-026-05204-7)
Supplement: Supplementary file 2 [file 425_2026_5204_MOESM1_ESM.docx]

**File S1** Plug-in MACRO script for ImageJ (FIJI)

//Blank ImageJ Macro Script that loops through files in a directory

// Specify global variables

input = getDirectory(&quot;Input_File&quot;);

output = input; // Output images to the same directory as input (prevents second dialogue box,

otherwise getDirectory(&quot;Output Directory&quot;))

Dialog.create(&quot;File Type&quot;);

Dialog.addString(&quot;File Suffix: &quot;, &quot;.JPG&quot;, 5); // Select another file format if desired

suffix = Dialog.getString();

processFolder(input);

// Scan folders/subfolders/files to locate files with the correct suffix

function processFolder(input) {

list = getFileList(input);

for (i = 0; i &lt; list.length; i++) {

if(File.isDirectory(input + list[i]))

processFolder(&quot;&quot; + input + list[i]);

if(endsWith(list[i], suffix))

processFile(input, output, list[i]);

}

}

// Loop through each file

function processFile(input, output, file) {

// Define all variables

MonthNames =

newArray(&quot;Jan&quot;,&quot;Feb&quot;,&quot;Mar&quot;,&quot;Apr&quot;,&quot;May&quot;,&quot;Jun&quot;,&quot;Jul&quot;,&quot;Aug&quot;,&quot;Sep&quot;,&quot;Oct&quot;,&quot;Nov&quot;,&quot;Dec&quot;); // Generate

month names

DayNames = newArray(&quot;Sun&quot;, &quot;Mon&quot;,&quot;Tue&quot;,&quot;Wed&quot;,&quot;Thu&quot;,&quot;Fri&quot;,&quot;Sat&quot;); // Generate date names

getDateAndTime(year, month, dayOfWeek, dayOfMonth, hour, minute, second, msec); // Get date

and time information

// Do something to each file

open(file);

run(&quot;Lab Stack&quot;);

run(&quot;Stack to Images&quot;);

selectWindow(&quot;L*&quot;);

selectWindow(&quot;b*&quot;);

selectWindow(&quot;L*&quot;);

close();

selectWindow(&quot;a*&quot;);

run(&quot;Invert&quot;);

run(&quot;Image Expression Parser (Macro)&quot;, &quot;expression=A*B&gt;=300 a=a* b=b*&quot;);

selectWindow(&quot;Parsed with A*B&gt;=300&quot;);

setAutoThreshold(&quot;Otsu dark&quot;);

//run(&quot;Threshold...&quot;);

setAutoThreshold(&quot;Otsu dark&quot;);

setOption(&quot;BlackBackground&quot;, true);

selectWindow(&quot;a*&quot;);

close();

selectWindow(&quot;b*&quot;);

close();

run(&quot;Convert to Mask&quot;);

run(&quot;Median...&quot;, &quot;radius=8&quot;);

saveAs(&quot;jpg&quot;, input+&quot;Image 1_&quot; + list[i] + &quot;.jpg&quot;);

run(&quot;Create Selection&quot;);

run(&quot;Measure&quot;);

saveAs(&quot;Results&quot;, input+&quot;Results&quot; + list[i] + &quot;.xls&quot;);

close();

// Print log of activities for reference...

print (DayNames[dayOfWeek], dayOfMonth, MonthNames[month], year + &quot;,&quot; + hour + &quot;:&quot; + minute +

&quot;:&quot; + second + &quot;: Processing &quot; + input + file);

}

// A final statement to confirm the task is complete...

print(&quot;Task complete.&quot;);
