# Supplementary material for: Light modulates organ-specific gibberellin–abscisic acid crosstalk during drought response in Arabidopsis thaliana
Source: Planta. 2026 Oct 17;264(6):172. doi: 10.1007/s00425-026-05204-7 (PMC13676654; doi:10.1007/s00425-026-05204-7)
Supplement: Supplementary file 3 [file 425_2026_5204_MOESM5_ESM.docx]

**Table S1** Primer sequences for RT-qPCR.

| **Gene** | **AGI number** | **5´-3´ primers** |
| --- | --- | --- |
| GA2ox2_F | AT1G30040 | ATTTTCCGTGAGTCGGTGGA |
| GA2ox2_R |  | CAGGCACGAGTCACTCTTCT |
| GA2ox6_F | AT1G02400 | TCAGGCATTGACGAATGGGA |
| GA2ox6_R |  | CTAACGGTGGAGCTGCAAAG |
| GA2ox9_F | AT3G47190 | GATATGATGCAGGCCATAAGC |
| GA2ox9_R |  | GTGTCTCTCTGTCTTCCTATTCC |
| GA20ox1_F | AT1G13320 | TTCACCAAGACCATGTTAATGGC |
| GA20ox1_R |  | TCGATAGAGCCATGAAAGTATCG |
| GA3ox1_F | AT1G15550 | ACCTCAACTACTGCGATATCG |
| GA3ox1_R |  | AATGTCTTCTTCGCTGACCC |
| GID1B_F | AT3G63010 | GGATCGGTTTGTTTTGCCTA |
| GID1B_R |  | TTATGCTTCCGGAGACCAAG |
| NCED3_F | AT3G14440 | CATCCTCTGTTTCGTTCACG |
| NCED3_R |  | TACGGAACCCTTGACGGAAG |
| PP2A_F | AT1G13320 | GTTCCAAACTCTTACCTGCGG |
| PP2A_R |  | GTTCTCCACAACCGCTTGGT |
| RD29a_F | AT5G52310 | GGGTAGAGATTATGTGGCGG |
| RD29a_R |  | CCTTTGTCGTCGTTTCCTTC |
