# Supplementary material for: Light modulates organ-specific gibberellin–abscisic acid crosstalk during drought response in Arabidopsis thaliana
Source: Planta. 2026 Oct 17;264(6):172. doi: 10.1007/s00425-026-05204-7 (PMC13676654; doi:10.1007/s00425-026-05204-7)
Supplement: Supplementary file 4 [file 425_2026_5204_MOESM8_ESM.docx]

|  |  |  | Col-0 |  |  | *aba2* |  |  | *ga2ox7/8* |  |  | *ga2ox9/10* | |  | *ga2oxQ* |  |  | *ga20ox1/2* | |  | 35S::GA20ox1 | | |
| --- | --- | --- | --- | --- | --- | --- | --- | --- | --- | --- | --- | --- | --- | --- | --- | --- | --- | --- | --- | --- | --- | --- | --- |
|  |  |  |  |  |  |  |  |  |  |  |  |  |  |  |  |  |  |  |  |  |  |  |  |
| Leaf temperature water WL | | | 23.05 | ± | 0.38 | 20.89 | ± | 0.27 | 22.82 | ± | 0.27 | 23.37 | ± | 0.20 | 23.57 | ± | 0.60 | 22.87 | ± | 0.29 | 23.33 | ± | 0.13 |
| Leaf temperature drought WL | | | 24.28 | ± | 0.16 | 21.82 | ± | 0.88 | 23.86 | ± | 0.30 | 24.32 | ± | 0.09 | 24.70 | ± | 0.21 | 22.84 | ± | 0.43 | 24.44 | ± | 0.15 |
| Leaf temperature water BL | | | 23.25 | ± | 0.12 | 21.46 | ± | 0.80 | 23.07 | ± | 0.19 | 23.24 | ± | 0.23 | 23.48 | ± | 0.40 | 22.42 | ± | 0.33 | 23.42 | ± | 0.46 |
| Leaf temperature drought BL | | | 23.98 | ± | 0.35 | 21.30 | ± | 0.24 | 23.65 | ± | 0.34 | 23.97 | ± | 0.20 | 24.12 | ± | 0.22 | 22.66 | ± | 0.27 | 24.27 | ± | 0.03 |
|  | | |  |  |  |  |  |  |  |  |  |  |  |  |  |  |  |  |  |  |  |  |  |

Temperature ± SD (°C)

*P-*values

| Comparison | Col-0 (WT) | *aba2* | *ga2ox7/8* | *ga2ox9/10* | *ga2oxQ* | *ga20ox1/2* | 35S::GA20ox1 |
| --- | --- | --- | --- | --- | --- | --- | --- |
| Water - drought WL | **7.37E-04** | 7.59E-02 | **4.44E-04** | **8.87E-05** | **1.06E-02** | 8.81E-01 | **1.54E-06** |
| Water - drought BL | **6.85E-03** | 6.90E-01 | **1.04E-02** | **6.76E-04** | **8.13E-03** | 2.42E-01 | **1.44E-02** |
| WL-BL water | 2.85E-01 | 1.90E-01 | 1.28E-01 | 3.74E-01 | 7.03E-01 | **4.91E-02** | 7.23E-01 |
| WL-BL drought | 1.37E-01 | 2.64E-01 | 3.40E-01 | **1.10E-02** | **2.75E-03** | 4.53E-01 | 5.11E-02 |
| Col-mutant water-WL* |  | **2.19E-04** | 1.0E+00 | 1.0E+00 | 1.0E+00 | 1.0E+00 | 1.0E+00 |
| Col-mutant water-BL* |  | 9.6E-02 | 1.0E+00 | 1.0E+00 | 1.0E+00 | 5.3E-02 | 1.0E+00 |
| Col-mutant drought-WL* |  | **4.0E-02** | 2.1E-01 | 6.2E-01 | 9.1E-02 | **1.5E-02** | 4.4E-01 |
| Col-mutant drought-BL* |  | **3.3E-05** | 8.9E-01 | 9.5E-01 | 9.5E-01 | **2.0E-03** | 8.9E-01 |
|  |  |  |  |  |  |  |  |
|  | | | |  |  |  |  |

**P*-values adjusted for multiple comparisons (Holm–Bonferroni method). Bold indicates *P*-values < 0.05. WL, white light; BL, blue light.

**Table S4** Leaf temperatures in watered and droughted Col-0, *aba2* and lines with altered GA metabolism growing in white or blue light. *P*-values for comparisons between watered and droughted plants, white and blue light and between Col-0 and other lines are also shown.
